# Supplementary material for: Long-term health and human capital effects of in utero exposure to an industrial disaster: a spatial difference-in-differences analysis of the Bhopal gas tragedy
Source: BMJ Open. 2023 May 9;13(6):e066733. doi: 10.1136/bmjopen-2022-066733 (PMC10335451; doi:10.1136/bmjopen-2022-066733)
Supplement: Supplementary data [file bmjopen-2022-066733supp001.pdf]

In Utero Exposure to Industrial Disasters: A Case Study of the Bhopal Gas Tragedy

Supplemental Material

Gordon C. McCord[1]

Prashant Bharadwaj[2]

Lotus McDougal[3]

Arushi Kaushik[4]

Anita Raj[5]

Figure S1 uses the Indian Human Development Survey (IHDS) in a robustness check to test whether mothers of those who were in utero at the time of the BGD are different from those of other cohorts. The IHDS-I was conducted in 2004-05 and is representative at the district level. It is the only other survey apart from the DHS that collects health information for India. We compare mothers' education level and several (self-reported) health parameters, such as the probability of reporting to be suffering from a heart disease, high blood pressure, diabetes, asthma, tuberculosis, leprosy, cancer and mental illness (n=1,792).

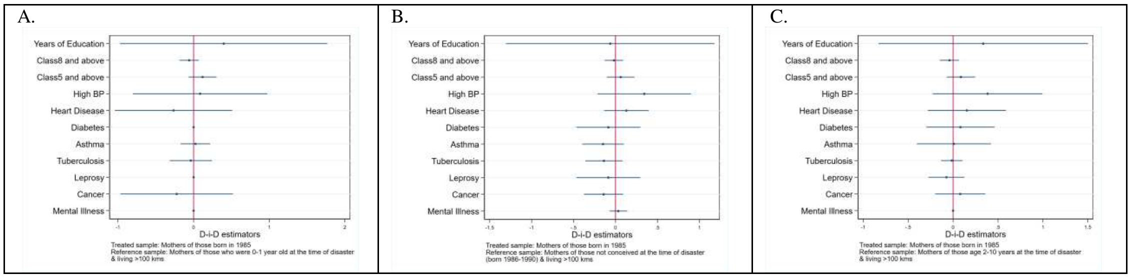

Figure S1: Characteristics of mothers of in utero cohort compared to others

The figure shows difference-in-difference estimates for various outcome variables for the mothers of those who were in utero (i.e, born in 1985). These estimates are obtained from regression equation similar to eq 1 where the control group includes mothers of non-mover men who were born in 1984 (i.e, < 1 year old at the time of BGD), Panel A; non-mover men who were born between 1986-1990 (i.e, not conceived at the time of BGD), Panel B; and non-mover men who were born between 1975-1983 (i.e, 2-10 years old at the time of BGD), Panel C; and are living > 100kms away from Union Carbide plant. The figures depict estimates with 95% CI.

[1] Corresponding author. gmccord@ucsd.edu, School of Global Policy and Strategy, UC San Diego
[2] Department of Economics, UC San Diego
[3] Center on Gender Equity and Health, Department of Medicine, UC San Diego
[4] Department of Economics, UC San Diego
[5] Center on Gender Equity and Health, Department of Medicine, UC San Diego

McCord GC, *et al. BMJ Open* 2023; 13:e066733. doi: 10.1136/bmjopen-2022-066733

Table S1: Linear probability models (LPM) for employment disability among men living in Madhya Pradesh in 1999 within 100km of Bhopal, compared to men of the same cohort more than 100km from Bhopal.

	Prob(Reported Employment Disability)		
	(1)	(2)	(3)
	Pooled	Non-Movers	Movers
1986-1990	-0.00109	-0.00131	0
	(0.00257)	(0.00297)	(5.63e-11)
1985	0.0111***	0.0115***	0
	(0.00216)	(0.00198)	(9.65e-11)
1975-1984	-0.000632	-4.65e-05	-0.00690
	(0.00344)	(0.00388)	(0.00608)
1960-1974	Reference	Reference	Reference
Observations	13,369	12,129	1,240
R-squared	0.003	0.003	0.024
Cohort FEs	Yes	Yes	Yes
Control Mean	0.00377	0.00426	0

Robust standard errors in parentheses

*** p<0.01, ** p<0.05, * p<0.1

Notes: The cohort of men born in 1985 within 100 km of Bhopal has a higher likelihood of reporting employment disability compared to other cohorts and to those born in Madhya Pradesh beyond 100 km from Bhopal (1), with effect most pronounced among men who have never moved (2) instead of those who have moved (3). Estimates are from ordinary least squares regressions as in Equation (I) with controls consisting of fixed effects for year of birth cohort and whether the DHS cluster is within 100 km of Bhopal. Standard errors are two-way clustered at the district and year-of-birth levels.

McCord GC, *et al. BMJ Open* 2023; 13:e066733. doi: 10.1136/bmjopen-2022-066733

Table S2: Linear probability models (LPM) for employment disability among men who have never moved living in Madhya Pradesh in 1999 at various distances from Bhopal, compared to men of the same cohort more than 100km from Bhopal.

	Prob(Employment Disability) among Non-Movers, by Treatment Radius		
	(1)	(2)	(3)
	100 km	75 km	50 km
1986-1990	-0.00131	-0.00183	-0.00045
	(0.00297)	(0.00390)	(0.00445)
1985	0.0115***	0.0138***	0.0203**
	(0.00198)	(0.00217)	(0.0078)
1975-1984	-4.65e-05	-0.00049	-0.00004
	(0.00388)	(0.00314)	(0.00294)
1960-1974	Reference	Reference	Reference
Observations	12,129	11,799	11,100
R-squared	0.003	0.002	0.003
Cohort FEs	Yes	Yes	Yes
Control Mean	0.00426	0.00437	0.0043

Robust standard errors in parentheses

*** p<0.01, ** p<0.05, * p<0.1

Notes: Estimates are from ordinary least squares regressions as in Equation (I) with controls consisting of fixed effects for year of birth cohort and whether the DHS cluster is within the specified distance from Bhopal. Standard errors are two-way clustered at the district and year-of-birth levels.

Table S3: Regression results on educational attainment among men born in Madhya Pradesh within 100km of Bhopal, compared to men of the same cohort more than 100km from Bhopal.

	Years of Education Completed		
	(1)	(2)	(3)
	Pooled	Non-Movers	Movers
1986-1990	0.221	-0.557	1.704*
	(0.601)	(0.897)	(0.851)
1985	-0.577	-2.276***	0.999*
	(0.376)	(0.714)	(0.557)
1975-1984	0.255	-0.0471	1.108
	(0.279)	(0.245)	(0.734)
1960-1974	Reference	Reference	Reference
Observations	7,045	5,014	2,031
R-squared	0.048	0.055	0.047
Cohort FEs	Yes	Yes	Yes
Control Mean	5.833	5.561	6.600

Robust standard errors in parentheses

*** p<0.01, ** p<0.05, * p<0.1

Notes: The cohort of men born in 1985 within 100 km of Bhopal has fewer years of educational attainment compared to other cohorts and to those born in Madhya Pradesh beyond 100 km from Bhopal (1), with effect most pronounced among men who have never moved (2) instead of those who have moved (3). Estimates are from ordinary least squares regressions as in Equation (I) with controls consisting of fixed effects for year of birth cohort and whether the DHS cluster is within 100 km of Bhopal. Standard errors are two-way clustered at the district and year-of-birth levels.

Table S4: Regression results on educational attainment among non-mover men born in Madhya Pradesh at varying distances from Bhopal, compared to other men of the same cohort beyond 100 km from Bhopal.

	Years of Education Completed among Non-Movers, by Treatment Radius		
	(1)	(2)	(3)
	100 km	75 km	50 km
1986-1990	-0.557	-0.356	-0.264
	(0.897)	(0.682)	(0.454)
1985	-2.276***	-2.253**	-0.316
	(0.714)	(0.983)	(2.200)
1975-1984	-0.0471	-0.371	-0.900
	(0.245)	(1.292)	(1.970)
1960-1974	Reference	Reference	Reference
Observations	5,014	4,830	4,702
R-squared	0.055	0.055	0.053
Cohort FEs	Yes	Yes	Yes
Control Mean	5.561	5.584	5.643

Robust standard errors in parentheses

*** p<0.01, ** p<0.05, * p<0.1

Notes: Estimates are from ordinary least squares regressions as in Equation (I) with controls consisting of fixed effects for year of birth cohort and whether the DHS cluster is within the specified distance from Bhopal. Standard errors are two-way clustered at the district and year-of-birth levels.

McCord GC, *et al. BMJ Open* 2023; 13:e066733. doi: 10.1136/bmjopen-2022-066733
