# Supplementary material for: Long-term health and human capital effects of in utero exposure to an industrial disaster: a spatial difference-in-differences analysis of the Bhopal gas tragedy
Source: BMJ Open. 2023 May 9;13(6):e066733. doi: 10.1136/bmjopen-2022-066733 (PMC10335451; doi:10.1136/bmjopen-2022-066733)
Supplement: Author's manuscript [file bmjopen-2022-066733.draft_revisions.pdf]

BMJ Open is committed to open peer review. As part of this commitment we make the peer review history of every article we publish publicly available.

When an article is published we post the peer reviewers' comments and the authors' responses online. We also post the versions of the paper that were used during peer review. These are the versions that the peer review comments apply to.

The versions of the paper that follow are the versions that were submitted during the peer review process. They are not the versions of record or the final published versions. They should not be cited or distributed as the published version of this manuscript.

BMJ Open is an open access journal and the full, final, typeset and author-corrected version of record of the manuscript is available on our site with no access controls, subscription charges or pay-per-view fees (http://bmjopen.bmj.com).

If you have any questions on BMJ Open's open peer review process please email info.bmjopen@bmj.com

BMJ Open

In Utero Exposure to Industrial Disasters: A Case Study of the Bhopal Gas Tragedy

Journal:	*BMJ Open*
Manuscript ID	bmjopen-2022-066733
Article Type:	Original research
Date Submitted by the Author:	18-Jul-2022
Complete List of Authors:	McCord, Gordon C ; University of California San Diego, School of Global Policy and Strategy<br>Bharadwaj, Prashant; University of California San Diego, Department of Economics<br>McDougal, Lotus; University of California San Diego, Department of Medicine<br>Kaushik, Arushi; University of California San Diego, Department of Economics<br>Raj, Anita; University of California San Diego, Department of Medicine
Keywords:	PUBLIC HEALTH, EPIDEMIOLOGY, HEALTH ECONOMICS

SCHOLARONE™
Manuscripts

I, the Submitting Author has the right to grant and does grant on behalf of all authors of the Work (as defined in the below author licence), an exclusive licence and/or a non-exclusive licence for contributions from authors who are: i) UK Crown employees; ii) where BMJ has agreed a CC-BY licence shall apply, and/or iii) in accordance with the terms applicable for US Federal Government officers or employees acting as part of their official duties; on a worldwide, perpetual, irrevocable, royalty-free basis to BMJ Publishing Group Ltd ("BMJ") its licensees and where the relevant Journal is co-owned by BMJ to the co-owners of the Journal, to publish the Work in this journal and any other BMJ products and to exploit all rights, as set out in our licence.

The Submitting Author accepts and understands that any supply made under these terms is made by BMJ to the Submitting Author unless you are acting as an employee on behalf of your employer or a postgraduate student of an affiliated institution which is paying any applicable article publishing charge ("APC") for Open Access articles. Where the Submitting Author wishes to make the Work available on an Open Access basis (and intends to pay the relevant APC), the terms of reuse of such Open Access shall be governed by a Creative Commons licence – details of these licences and which Creative Commons licence will apply to this Work are set out in our licence referred to above.

Other than as permitted in any relevant BMJ Author's Self Archiving Policies, I confirm this Work has not been accepted for publication elsewhere, is not being considered for publication elsewhere and does not duplicate material already published. I confirm all authors consent to publication of this Work and authorise the granting of this licence.

For peer review only

In Utero Exposure to Industrial Disasters: A Case Study of the Bhopal Gas Tragedy

Gordon C. McCord[1]

Prashant Bharadwaj[2]

Lotus McDougal[3]

Arushi Kaushik[4]

Anita Raj[5]

Abstract

Objectives: Globalization and industrialization can increase economic opportunity for low- and middle-income nations, but these processes may also increase industrial accidents and harm workers. This paper examines the long-term, cohort-based health effects of the Bhopal Gas Disaster (BGD), one the most serious industrial accidents in history.

Design: This retrospective analysis uses geolocated data on health and education from India's National Family Health Survey-4 (NFHS-4) and the 1999 Indian Socio-Economic Survey [NSSO-1999] to examine the health effects of exposure to the BGD among men and women aged 15-49 living in Madhya Pradesh in 2015-16 (women n=40,786; men n=7,031 [NFHS-4] and n=13,369 [NSSO-1999]), as well as their children (n=1,260). A spatial difference-in-differences strategy estimated the relative effect of being in utero near Bhopal relative to other cohorts and to those further from Bhopal separately for each dataset.

Results: We document long-term, intergenerational impacts of the BGD, showing that men who were in utero at the time were more likely to have a disability that affected their employment 15 years later, and had higher rates of cancer and lower educational attainment over 30 years later. Changes in the sex ratio among children born in 1985 suggest an effect of the BGD up to 100km from the accident.

Conclusions: These results indicate social costs stemming from the BGD that extend far beyond the mortality and morbidity experienced in the immediate aftermath. Quantifying these multigenerational impacts are important for policy consideration. Moreover, our results suggest that the BGD affected people across a substantially more widespread area than has previously been demonstrated.

Ethical approval: All data used in this study is publicly available and de-identified. Ethical approval for NFHS-4 data collection was provided by the International Institute for Population Sciences and ICF. Ethical approval for the 1999 NSSO Socio-Economic Survey was provided by the Indian National Sample Survey Office. Ethical exemption for this analysis was provided by the University of California San Diego IRB.

Transparency statement: The authors affirm that the manuscript is an honest, accurate, and transparent account of the study being reported; that no important aspects of the study have been omitted.

Funding*:* AR received funding from the Bill and Melinda Gates Foundation (OPP1179208).

[1] Corresponding author. gmccord@ucsd.edu, School of Global Policy and Strategy, UC San Diego
[2] Department of Economics, UC San Diego
[3] Center on Gender Equity and Health, Department of Medicine, UC San Diego
[4] Department of Economics, UC San Diego
[5] Center on Gender Equity and Health, Department of Medicine, UC San Diego

For peer review only - http://bmjopen.bmj.com/site/about/guidelines.xhtml

Summary Box

What is already known on this topic

- Findings from an exhaustive review of the peer-reviewed published literature on the Bhopal Gas Disaster (BGD) brought out important research documenting immediate and longer-term health consequences of those residing in the affected area.
- Less research has focused on these longer-term effects on those in utero and those who were young children at the time of the BGD, despite demonstrated effects on menstruation and miscarriage among adult women exposed to the BGD.

What this study adds

- This study is also the first, to our knowledge, to use different birth cohorts to attribute causality of subsequent health and social consequences to the BGD for those in utero or very young at time of exposure.
- We document long-term, intergenerational impacts of the BGD, showing that men who were in utero at the time were more likely to have a disability that affected their employment 15 years later, and had higher rates of cancer and lower educational attainment over 30 years later.
- The evidence starkly highlights the long-term, intergenerational health and human capital effects of the BGD, and underscores the need for ongoing survivor support, as well as robust regulatory protections.

Introduction

Globalization and industrialization can increase economic opportunity for low- and middle-income nations, but recent cross-national evidence indicates that these processes may also be increasing industrial accidents and harming the workers of these nations.[1] Evidence from India suggests high numbers of industrial accidents, with the highest associated attributable deaths in the world.[2 3] Despite recommendations for improved protections for workers and industry in India,[3] the Ministry of Environment, Forest and Climate Change has proposed the Environmental Impact Assessment (EIA) notification 2020, which will roll back environmental and worker protections in the country. Critics, including United Nations experts, have stated that this rollback violates the safety and health of workers and the environment and with potential impact on subsequent generations.[4-6] Unfortunately, little research is available on these multigenerational impacts, and inadequate recognition of these far-reaching repercussions can diminish the memory of such tragedies for policy consideration. This research provides insight into these impacts for children born to women survivors of one of the worst industrial disasters in history, the Bhopal Gas Disaster.

The Bhopal Gas Disaster (BGD) occurred in December 1984 and involved a methyl isocyanate (MIC) gas leak at a Union Carbide pesticide plant near the city of Bhopal, in Madhya Pradesh, India. The leak spread toxic gas in approximately a 7 km radius around the plant, exposing more than half a million people and resulting in up to 30,000 deaths in the region.[7] There is a broad spectrum of serious long term and chronic health consequences for hundreds of thousands of survivors, including children, manifesting across multiple systems (e.g. respiratory, neurologic, musculoskeletal, ophthalmic, endocrine).[7-10] These impacts may be the tip of the iceberg however, given that MIC toxins affected groundwater[11] and the reproductive health and other health outcomes of exposed women,[8] factors suggesting generations not exposed to the toxic gas directly may nevertheless suffer adverse health and social impacts. Potential in utero effects cannot be understated. Studies document a 4-fold increase in the rate of miscarriage following the gas leak, as well as increased risk for stillbirth and neonatal mortality.[9 10] Decades after the

For peer review only - http://bmjopen.bmj.com/site/about/guidelines.xhtml

disaster, menstrual abnormalities and premature menopause have emerged as common problems among exposed women and their female offspring.[9] MIC has also been shown to damage human chromosomes.[12] Early clinical studies on the gas-affected population showed signs of increased chromosomal aberrations that could manifest as cancer,[13] though population-level changes in cancer rates 8 years after the accident were suggestive but not significant.[14]

This paper estimates the long-term health (specifically, adult cancer rates and disability) and human capital impacts (educational attainment) on individuals who were exposed to the BGD in utero or as children in 1984. Understanding the long-term impacts of industrial disasters on children, especially those in utero at the time of the disaster, is important for several key reasons. First, while the immediate damage to children in the form of direct health impacts is perhaps easier to assess, the long term, health related damages could take years to manifest and might not be part of the legal damages considered. Second, economic research over the last few decades has shown that interventions that improve outcomes for children have high rates of return;[15] to this second point, we include education as an important indicator of socioeconomic impact on these children, particularly as this may compound health impacts. Our findings offer timely information for consideration of potential rollbacks of policies related to social and environmental protections attached to industrialization in India and elsewhere.

Methods

Data

The principal data source is the fourth round of the Demographic and Health Surveys (DHS) in India, which was conducted between 2015-2016 (also called National Family Health Survey, or NFHS-4, in India).[16] The DHS/NFHS-4 is a high-quality household survey that provides detailed information on health at the individual level, and is representative at both the national and state levels. All interviews in Madhya Pradesh were completed in 2015. For the first analysis focusing on differences in the sex ratio, the analytic sample includes children born between 1981-1985 to women who were interviewed in Madhya Pradesh and who lived within 250 km of Bhopal (n=1,260 births with nonmissing data on child sex). The analytic sample for the analysis of cancer incidence includes men and women interviewed in Madhya Pradesh and lived within 250 km of Bhopal, and were born between 1960 and 1990 (n=7,031 men and 40,786 women).

A second dataset that we use is the Integrated Public Use Microdata Series (IPUMS) from India for the year 1999, which presents harmonized data from the 1999 Socio-Economic Survey conducted by the National Sample Survey Organization (NSSO).[17] These data contain employment information for a representative sample but only identifies the district of the respondent. This is the most recent NSSO survey in which respondents were asked how long they had been living at their current residence, a crucial question for this analysis. The survey sample includes people from ages 6-64, thus including those who were in-utero at the time of the BGD. The analytic sample for this analysis includes men interviewed in Madhya Pradesh and born between 1960 and 1990 (n=13,369)

These two datasets could not be merged at the individual level, as the same respondents were not interviewed in each survey. In order to preserve the more precise geolocation of the NHFS-4, the two datasets were analyzed separately.

Patient and Public Involvement
Since DHS data are anonymized, direct involvement with study subjects is not possible. Once published, results will be disseminated to the public in Bhopal through a letter to local newspaper media.

For peer review only - http://bmjopen.bmj.com/site/about/guidelines.xhtml

Measures

For the analysis of children born to women surveyed by the NFHS-4, the outcome of interest was the ratio of male-to-female births.

For men, the outcomes of interest were cancer rates and educational attainment (from the NFHS-4) and employment disability (from the NSSO). Cancer was assessed as a general question "Do you currently have cancer", and educational attainment was assessed as the number of years that an individual attended school. Of the sample of men, 17 out of 7,031 with nonmissing response reported to have cancer, and the average years of education was 7.0 with a standard deviation of 5.1. We use the question on whether "the respondent was economically inactive because of disabilities or...other health-related reasons" to classify the person as suffering from employment disability. 46 in the sample of 13,369 report to be suffering from employment disability. Given the prevalence of child labor, note that the employment-related questions of the survey are asked of all respondents, including children. Analyses assessing movers vs. non-movers are necessarily restricted to men, since patrilocality in India means that most women move to their husband's house after marriage.[18] As 78% of Indian women aged 15-49 have married,[16] the sample size of non-mover females is both small and non-representative, and thus cannot be considered in this analysis.

For all analyses, distance from the Union Carbide plant was the primary predictor. The NFHS-4 collected the latitude and longitude of each respondent's sampling cluster (henceforth, DHS clusters). The DHS clusters are villages or hamlets in rural areas and blocks or census tracts in urban areas; thus, they are much smaller geographical units than administrative units such as districts. These coordinates enable the calculation of distance from the Union Carbide plant in Bhopal for each respondent, within a privacy displacement radius of 2 km (urban) or 5-10 km (rural) (Figure 1).

Figure 1: Madhya Pradesh and DHS cluster locations around Bhopal

In the case of the district-level NSSO data, we measure distance from the Union Carbide plant to the nearest border of the district. For both datasets, we use the distance from the plant to form the treatment and control groups, since exposure to the toxic MIC gas would be more muted at greater distances.

Analysis

The analysis uses a spatial difference-in-differences strategy, where distance from the Union Carbide plant (we also refer to this as "distance from Bhopal") lends the spatial dimension. We compare people living close to Bhopal to those far away from Bhopal, and people who were young at the time of the BGD to those who were older at the time of BGD. Under some standard assumptions, these estimates net out any difference in outcomes that arise due to *time invariant* aspects of being close to Bhopal, as well as aspects of belonging to a specific birth cohort.

All analyses were conducted using birth year cohorts (Figure 2). The reference cohort includes those born between 1960-1974 (i.e. respondents who were more than 10 years old at the time of the BGD). Three other cohorts are considered: young children below 10 years old (i.e., respondents born between 1975-1984), fetuses (i.e. respondents born in 1985) and children not yet conceived at the time of BGD (i.e. respondents born between 1986-1990).

For peer review only - http://bmjopen.bmj.com/site/about/guidelines.xhtml

Figure 2: Description of all cohorts used in empirical estimations.

It is worth noting that all four cohorts have been affected by the disaster, including our reference cohort. Therefore, while older cohorts are being used as a reference category, our results speak to the *relative* impacts on those who were in utero at the time of the disaster.

Linear regression models were used to estimate the relationship between proximity to Bhopal, birth cohort, and outcomes of interest, with the basic specification as follows:

$$\text{(I)} \qquad Y_{itc} = \alpha_1 Bhopal_c + \beta_1 {*} T1_t {*} Bhopal_c + \beta_2 {*} T2_t {*} Bhopal_c + \beta_3 {*} T3_t {*} Bhopal_c + \text{Cohort FE} + \varepsilon_{itc}$$

where Y_{itc} is the outcome of interest for person *i* born in year *t* and living in location *c*. We consider three health and education outcomes, namely: the probability of suffering from cancer, the probability of suffering from employment disability, and years of education completed. $Bhopal_c$ takes the value of 1 if the respondent *i* is living near Bhopal (we define "near" Bhopal as being within 100kms following spatial patterns in sex ratios explained in Figure 3) and 0 otherwise. $T1_t$ is a dummy variable equal to 1 if the respondent was born between 1975-1984 (cohort of ages 0-10 at the time of BGD) and 0 otherwise. $T2_t$ is a dummy variable equal to 1 if the respondent was born in 1985 (in-utero cohort) and 0 otherwise. $T3_t$ is a dummy variable equal to 1 if the respondent was born between 1986-1990 (cohort not yet conceived at the time of BGD) and 0 otherwise. The omitted category consists of respondents who were older than 10 years at the time of the disaster and are living more than 100 kms away from Bhopal. All regressions include birth year fixed effects to standardize the outcome variable across ages. Standard errors are two-way clustered by birth year and district in order to account for spatial and serial autocorrelation.

β_1, β_2, and β_3 respectively capture the effect of BGD on young children below 10 years old, fetuses and not-yet-conceived children relative to those who were older than 10 at the time of BGD. The spatial difference-in-difference compares children in a cohort living near Bhopal to children in the same cohort living far from Bhopal. In order to interpret β_1, β_2, and β_3 as the causal effect of the BGP, the underlying identifying assumption is that in the absence of the disaster the cohorts near and far from Bhopal would have evolved similarly. Note that α_1 captures the effect of living near Bhopal vis-a-vis far from Bhopal for the reference cohort (1965-1974). This difference captures time invariant differences in the two geographical locations.

Results

Sex ratio changes following the Bhopal Gas Disaster

The sex ratio of children born in 1981-1984 is markedly different from that of children born in 1985 (Figure 3). Women who lived within 100 km of Bhopal experienced a relative decrease in the birth of males in the 1985 cohort (64% of children born from 1981-1984 were male, a proportion that drops to 60% in 1985). This is consistent with male fetuses more affected by external stress.[19-21] Women living beyond 100km had no difference in the sex ratio across the 1981-1984 and the 1985 cohorts.

Figure 3. Difference in male-female sex ratio between 1985 and 1981-1984 cohorts among children born to women ages 15-49 in Madhya Pradesh, by distance to Union Carbide plant.

For peer review only - http://bmjopen.bmj.com/site/about/guidelines.xhtml

Adult Cancer Rates among Exposed Children

Similarly to patterns seen with changes in the sex ratio, children who were born in 1985 and who lived close to Bhopal during the disaster experience much higher rates of cancer as adults compared to adults who were born before or after the disaster and who lived further away from Bhopal (Figure 4). As in the case of differences in sex ratios, the abnormally high cancer rates appear to extend up to 100 km from Bhopal. Given the two spatial patterns in Figures 3 & 4, we use 100 km as the BGD exposure cutoff in our analysis.

Figure 4. Self-reported cancer incidence among men and women living in Madhya Pradesh in 2015, by distance to Union Carbide plant.

Among all men in the NFHS-4 analytic sample, estimates suggest that the 1975-1984 and 1986-1990 birth cohorts have statistically indistinguishable cancer rates compared to the 1960-1974 cohort, while men born within 100 km of Bhopal in 1985 have a 2.1 percentage point higher risk than the other cohorts (Table 1). This is an 8-fold higher cancer risk compared to the other cohorts.

Table 1: Probability of Suffering from Cancer among men born in Madhya Pradesh.

	Prob(Reports having Cancer)		
	(1)	(2)	(3)
	Pooled	Non-Movers	Movers
1986-1990	-0.00373	0.00170	-0.0141
	(0.00340)	(0.00147)	(0.0121)
1985	0.0209***	0.0624***	-0.00906
	(0.00591)	(0.00505)	(0.0113)
1975-1984	-0.00459	-0.00157	-0.00906
	(0.00355)	(0.00181)	(0.0113)
1960-1974	Reference	Reference	Reference
Observations	7,031	5,002	2,029
R-squared	0.007	0.012	0.014
Cohort FEs	Yes	Yes	Yes
Control Mean	0.00252	0.00228	0.00321

Robust standard errors in parentheses
*** p<0.01, ** p<0.05, * p<0.1

Among men who have never moved, and were therefore residing within 100km of the Union Carbide plant during the BGD, the effect of being born in 1985 and near Bhopal is even stronger. These men have a 6.2 percentage point higher cancer risk in 2015 compared to the other cohorts and to those living more than 100 km from Bhopal. This represents a 27-fold higher risk of cancer among adults who were in utero during the BGD, suggesting long-term health consequence of exposure, even while in the womb. Men who moved at some point before the survey in 2015/16 (and who were therefore less likely to be exposed to the BGD), and had no difference in cancer rates across cohorts.

For peer review only - http://bmjopen.bmj.com/site/about/guidelines.xhtml

Effects on Employment Disability

Men who were in utero during the BGD and who lived in districts with a border within 100 km Bhopal are 1 percentage point more likely to report employment disability when they are surveyed as adults, compared to the older cohorts and those living further from Bhopal (Figure 5; Table S1).

Figure 5: Employment disability, compared to reference group of men born from 1960-1974 and living > 100 km from Bhopal. Bars represent 95% confidence intervals. Note that there were no individuals reporting employment disability in the 1985 and 1986-1990 cohorts among the *movers* subgroup.

Effects on Educational Attainment

Relative to other cohorts, men who were in utero during the BGD attained approximately two fewer years of education, with the effects only evident among men who have never moved (Figure 6; Table S2).

Figure 6: Years of education completed, compared to reference group of men born from 1960-1974 and living > 100 km from Bhopal.

Discussion

The Bhopal Gas Disaster was one the most serious industrial accidents in history, causing thousands of deaths at the time of the leak, and tens of thousands more deaths and serious health sequelae in the three decades since.[22 23] This analysis documents long-term, intergenerational impacts of the BGD, showing that men who were in utero at the time of the BGD were more likely to have a disability that affected their employment 15 years later, and had higher rates of cancer and lower educational attainment over 30 years later. Importantly, these results indicate social costs stemming from the BGD that extend far beyond the mortality and morbidity experienced in the immediate aftermath. Moreover, our results suggest that the BGD affected people across a substantially more widespread area than has previously been demonstrated: up to around 100 km (approximately 60 miles) from the site, as opposed to the 4.5 km radius that was considered exposed by public health officials and researchers after the disaster.[9 10 14 24] This study is also the first, to our knowledge, to use different birth cohorts to attribute causality of subsequent health and social consequences to the BGD.

We find that men currently living within 100 km of Bhopal and born in 1985 have an 8-fold higher risk of cancer than men of other birth cohorts; of those men, those who have never changed residence since the BGD have a 27-fold higher risk of cancer. While elevated cancer levels have been previously associated with MIC exposure,[14 25-27] our results identify long-term, birth-year based differences in cancer prevalence. This underscores the plausibility of multiple MIC exposure pathways, including teratogenic in utero exposure, and ongoing environmental exposure. Bhopal has ongoing cancer surveillance programs,[28] and it remains clear that those exposed to MIC require ongoing and careful monitoring, support and treatment.

Employment disability was one percentage point more likely among men who were in utero in districts within 100 km of Bhopal during the BGD than those born prior or more distal to the BGD. This is a meaningful impact since baseline employment disability rates are quite low (0.4%), and men's employment at the time of study was nearly universal at 98%.[16] In a context of high poverty, with nearly

For peer review only - http://bmjopen.bmj.com/site/about/guidelines.xhtml

40% of Madhya Pradesh's population living beneath the poverty line,[29] more than 90% informal employment nationally (thus lacking social protections and safety nets),[30] and household reliance on male income due to low levels of female labor force participation and a highly skewed gender wage gap,[16 31] this increase in disability or health-related economic inactivity risks substantial impact on the health, safety and well-being of affected families.

The finding that men who were in utero and within 100km of Bhopal during the BGD received more than two fewer years of education than other cohorts has tremendous human capital implications. This is a large impact both since the average number of years of education in the control group is only 5.6 years, and because education has such a direct association with subsequent wages and consumption.[32] We cannot ascertain whether the lower educational attainment is due to health or cognitive consequences of being exposed to the BGD in utero. Given that the BGD effect is much more evident in the 1985 cohort compared to older or younger children, it is unlikely that the effect operated through broader socioeconomic impacts or impacts on other family members that limited children's access to school. Regardless of the mechanism, the reduced educational attainment is evidence of long-term consequences of exposure to the accident that imply personal and social costs far beyond the immediate health impacts.

A few study limitations are worth outlining. First, as with any ecological exposure assignment, the cohort of in-utero children we assign to exposure will include a range of actual exposure to MIC gas. The interpretation of our estimates would be the effect of average exposure in the population we define as treated. Secondly, our estimates are affected by mortality and migration. An important consequence of a disaster, whether natural or man-made, is that mortality can severely affect the composition of the population observed post-facto. For example, if the weakest children in utero were more likely to die from the incident, then our estimates could be considered a lower bound. In our empirical context, any mortality effect of the disaster that symmetrically affects all cohorts within Bhopal (without affecting people away from Bhopal) will not affect our estimates. As such, it is differential mortality across cohorts due to the BGD that would have compositional effects on our study sample.

Likewise, a frequent human response to a disaster is migration to safer areas. Again, it would only be differential migration in response to the BGD (different migration across cohorts and across people living close or far from Bhopal) that would create a problematic compositional difference between our treated cohort and the others. In fact, migration in this area was relatively low and researchers found that 91% of the population remained in the same area subsequent to 1984.[10] Our own estimates in Figure S1 suggest that mothers of the in-utero cohort are not significantly different from mothers of other consecutive cohorts on various educational and health dimensions.

It is also important to note that the long-term consequences that we estimate could be the result of both direct effects from exposure as well as lack of subsequent mitigation of the effects through health, disability and education services. While disentangling these forces is important from a policy perspective, in this paper we simply highlight the total combined effect of being exposed to such disasters as a child. Finally, cancer reporting in the data comes from self-reports, which could be subject to biases; there are also very cases of self-reported cancer in the data. We think of our results on cancer as being consistent with the broader public health narrative on the consequences of the disaster, but more data through systematic cancer screening and place of birth information is key to firmly establish this link.

Understanding the short- and long-term damages caused by industrial disasters is key to gaining insight into the tradeoffs involved in making regulatory decisions. It is also crucial from a policy response perspective if policy makers wish to dedicate resources to mitigate harm done by such events or to compute legal damages. These concerns are particularly germane now, when there is evidence that the multiple health conditions suffered by many BGD survivors may make them more susceptible to the COVID-19 pandemic currently ravaging India.[33 34] The evidence presented in this paper starkly highlights

For peer review only - http://bmjopen.bmj.com/site/about/guidelines.xhtml

the long-term, intergenerational health and human capital effects of the BGD, and underscores the need for ongoing survivor support, as well as robust regulatory protections.

Funding Statement

AR received funding from the Bill and Melinda Gates Foundation (OPP1179208).

Declaration of interests

The authors declare no conflict of interest.

Author contributions

PB conceptualized the project, GCM and AK organized data and conducted analysis, all authors contributed equally to data interpretation, writing, review and editing. GCM and AK have verified the underlying data.

Ethics Statement

All data used in this study is publicly available and de-identified. Ethical approval for NFHS-4 data collection was provided by the International Institute for Population Sciences and ICF. Ethical approval for the 1999 NSSO Socio-Economic Survey was provided by the Indian National Sample Survey Office. Ethical exemption for this analysis was provided by the University of California San Diego IRB.

Data Sharing Statement

Replication code will be made available upon publication. The raw DHS survey data are subject to a user agreement and are available at available from the Demographics and Health Surveys Program at https://dhsprogram.com. The IPUMS data with the 1999 National Sample Survey are available from https://international.ipums.org/international/index.shtml.

The Corresponding Author has the right to grant on behalf of all authors and does grant on behalf of all authors, an exclusive licence (or non exclusive for government employees) on a worldwide basis to the BMJ Publishing Group Ltd to permit this article (if accepted) to be published in BMJ editions and any other BMJPGL products and sublicences such use and exploit all subsidiary rights, as set out in our licence.

For peer review only - http://bmjopen.bmj.com/site/about/guidelines.xhtml

For peer review only

References

1. Blanton RG, Peksen D. Dying for globalization? The impact of economic globalization on industrial accidents. *Social Science Quarterly* 2017;98(5):1487-502.
2. Nalapat S. Workplace disasters during lockdown put spotlight on India's patchy safety record -- explained with graphs. *Times Now News* 2020 September 7, 2020.
3. Sengupta A, Bandyopadhyay D, Van Westen C, et al. An evaluation of risk assessment framework for industrial accidents in India. *Journal of loss prevention in the process industries* 2016;41:295-302.
4. Explained: What is EIA 2020? How does it water down the existing policy? *The Week* 2020 August 10, 2020.
5. Explained Ideas: What ails with the draft EIA notification 2020? *The Indian Express* 2020 August 12, 2020.
6. UN experts to Govt of India: Environmental exemptions to industry unwarranted. 2020 September 4, 2020.
7. Sharma DC. Bhopal: 20 years on. *Lancet* 2005;365(9454):111-2. doi: 10.1016/S0140-6736(05)17722-8 [published Online First: 2005/01/12]
8. Ganguly BB, Mandal S, Kadam NN. Spectrum of health condition in methyl isocyanate (MIC)-exposed survivors measured after 30 years of disaster. *Environ Sci Pollut Res Int* 2018;25(5):4963-73. doi: 10.1007/s11356-017-0865-6 [published Online First: 2017/12/06]
9. Mishra PK, Samarth RM, Pathak N, et al. Bhopal Gas Tragedy: review of clinical and experimental findings after 25 years. *Int J Occup Med Environ Health* 2009;22(3):193-202. doi: 10.2478/v10001-009-0028-1 [published Online First: 2009/10/13]
10. Dhara VR, Dhara R. The Union Carbide disaster in Bhopal: a review of health effects. *Archives of Environmental Health: An International Journal* 2002;57(5):391-404.
11. Mishra B, Banerjee N. MIC leak disaster and environmental contamination: Time to act now. *Natl J Community Med* 2014;5:13-20.
12. Ennever FK, Rosenkranz HS. Evaluating the potential for genotoxic carcinogenicity of methyl isocyanate. *Toxicol Appl Pharmacol* 1987;91(3):502-05.
13. Malla T, Senthilkumar C, Sharma N, et al. Chromosome instability among Bhopal gas tragedy survivors. *Am Eur J Toxicol Sci* 2011;3:245-9.
14. Dikshit RP, Kanhere S. Cancer patterns of lung, oropharynx and oral cavity cancer in relation to gas exposure at Bhopal. *Cancer Causes Control* 1999;10(6):627-36.
15. Magnuson K, Duncan GJ. Can early childhood interventions decrease inequality of economic opportunity? *RSF: The Russell Sage Foundation Journal of the Social Sciences* 2016;2(2):123-41.
16. International Institute for Population Sciences (IIPS), ICF. National Family Health Survey (NFHS-4), 2015-16: India. Mumbai: IIPS, 2017.
17. National Sample Survey Office - M/o Statistics and Implementation (MOSPI), Government of India. India - Informal Non-Agricultural Enterprises Survey, July 1999 - June 2000, NSS 55th Round. In: NSSO, ed. Delhi, India, 1999-2000.
18. Khalil U, Mookerjee S. Patrilocal Residence and Women's Social Status: Evidence from South Asia. *Economic Development and Cultural Change* 2019;67(2):401-38. doi: 10.1086/697584
19. Kraemer S. The fragile male. *BMJ (Clinical research ed)* 2000;321(7276):1609-12. doi: 10.1136/bmj.321.7276.1609
20. Hansen D, Moller H, Olsen J. Severe periconceptional life events and the sex ratio in offspring: follow up study based on five national registers. *BMJ (Clinical research ed)* 1999;319(7209):548-49. doi: 10.1136/bmj.319.7209.548
21. Mizuno R. The male/female ratio of fetal deaths and births in Japan. *Lancet* 2000;356(9231):738-9. doi: 10.1016/s0140-6736(00)02637-4
22. Broughton E. The Bhopal disaster and its aftermath: a review. *Environmental Health* 2005;4(1):6. doi: 10.1186/1476-069X-4-6

For peer review only - http://bmjopen.bmj.com/site/about/guidelines.xhtml

23. Odysseos L. The question concerning human rights and human rightlessness: disposability and struggle in the Bhopal gas disaster. *Third World Quarterly* 2015;36(6):1041-59.
24. Sathyamala C, Jayaprakash N. The Bhopal Disaster and Medical Research. *Economic and Political Weekly* 2012:37-40.
25. Khan I, Senthilkumar CS, Upadhyay N, et al. In silico docking of methyl isocyanate (MIC) and its hydrolytic product (1, 3-dimethylurea) shows significant interaction with DNA Methyltransferase 1 suggests cancer risk in Bhopal-Gas-Tragedy survivors. *Asian Pacific Journal of Cancer Prevention* 2015;16(17):7663-70.
26. Senthilkumar CS, Malla TM-u-D, Sah NK, et al. Cancer Morbidity Among Methyl Isocyanate Exposed Long-Term Survivors and Their Offspring: A Hospital-Based Five Year Descriptive Study (2006-2011) and Future Directions to Predict Cancer Risk in the Affected Population. *Asian Pacific journal of cancer prevention: APJCP* 2011;12(12):3443-52.
27. Senthilkumar CS, Akhter S, Malla TM, et al. Increased micronucleus frequency in peripheral blood lymphocytes contributes to cancer risk in the methyl isocyanate-affected population of Bhopal. *Asian Pac J Cancer Prev* 2015;16(10):4409-19. doi: 10.7314/apjcp.2015.16.10.4409 [published Online First: 2015/06/02]
28. National Centre for Disease Informatics and Research. Area Cancer National Cancer Registry Programme, India; 2021 [Available from: https://ncdirindia.org/Area_Cancer.aspx accessed June 8 2021.
29. Ministry of Social Justice and Empowerment, Department of Social Justice and Empowerment, Government of India. State-Wise Percentage of Population Below Poverty Line by Social Groups, 2004-05: Government of India; 2021 [Available from: http://socialjustice.nic.in/UserView/index?mid=76672 accessed June 8 2021.
30. Mehrotra S. Informal Employment Trends in the Indian Economy: Persistent informality, but growing positive development. In: Employment Policy Department I, ed. Employment: Working Paper No 254. Geneva, Switzerland: International Labour Organisation, 2019.
31. ILO Decent Work Team for South Asia and Country Office for India. India Wage Report: Wage policies for decent work and inclusive growth. Delhi, India: International Labour Organization, 2018.
32. Fulford S. Returns to education in India. *World Development* 2014;59:434-50.
33. Malviya A, Ahirwar AK, Chandra Tripathi S, et al. COVID-19: a review on SARS-CoV-2 origin, epidemiology, virology, clinical manifestations and complications with special emphasis on adverse outcome in Bhopal Gas Tragedy survivor. *Horm Mol Biol Clin Investig* 2021;42(1):63-68. doi: 10.1515/hmbci-2020-0070 [published Online First: 2021/02/19]
34. Dore B. Covid-19: collateral damage of lockdown in India. *BMJ* 2020;369:m1711. doi: 10.1136/bmj.m1711 [published Online First: 2020/05/02]

For peer review only

For peer review only - http://bmjopen.bmj.com/site/about/guidelines.xhtml

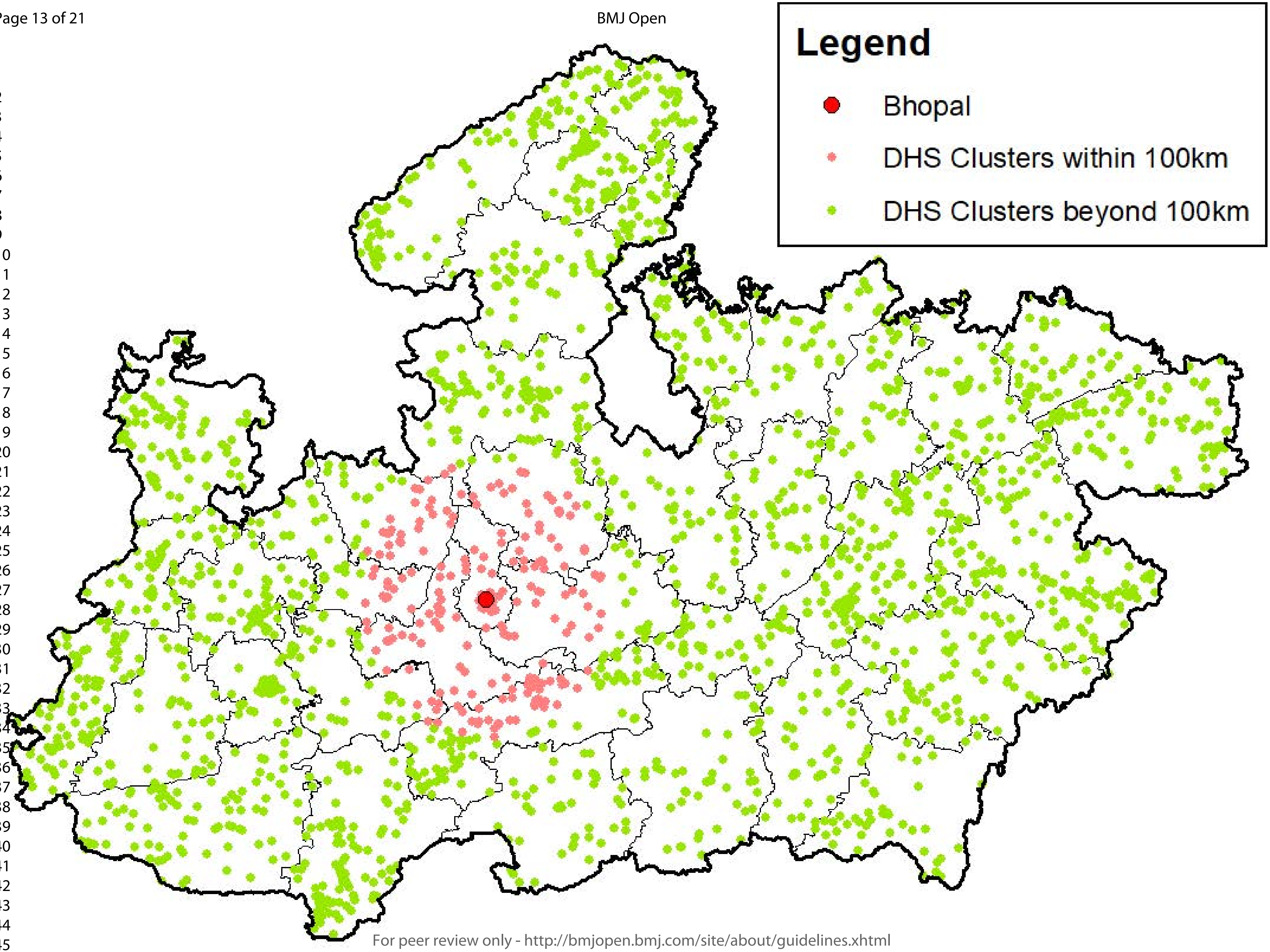
Legend
Bhopal
DHS Clusters within 100km
DHS Clusters beyond 100km

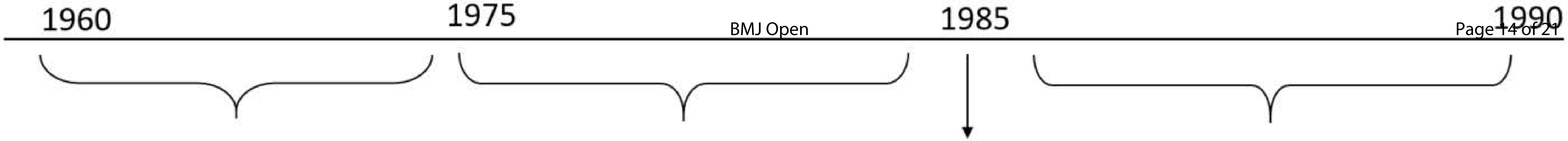

1960-1974	1975-1984	1985	1986-1990
Reference Cohort	T1	T2	T3
>10 years old at the time of Bhopal tragedy	0-10 years old at the time of Bhopal tragedy	Fetuses at the time of Bhopal tragedy	Not yet conceived at the time of Bhopal tragedy

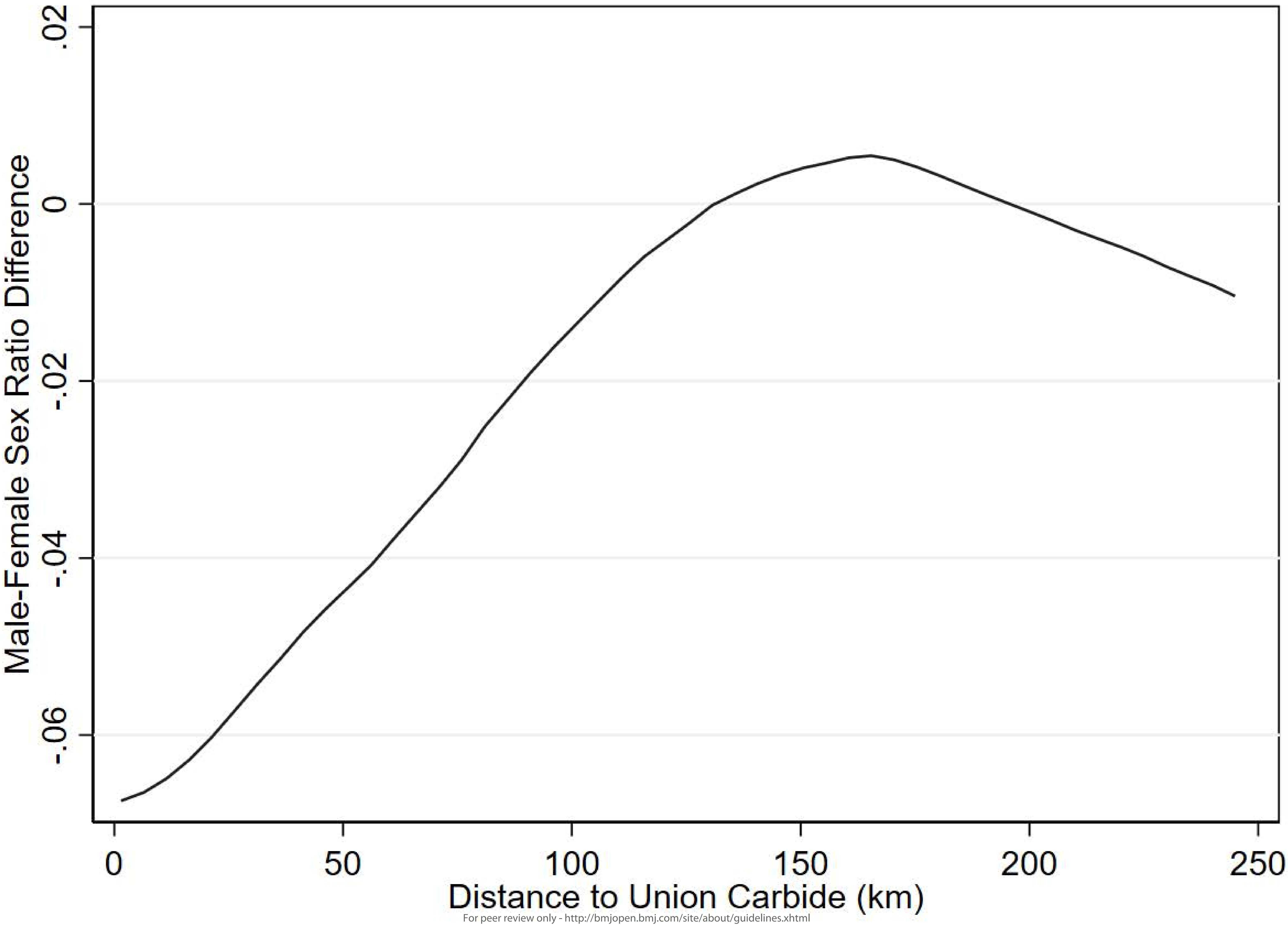
Male-Female Sex Ratio Difference
.02
0
-.02
-.04
-.06
0
50
100
150
200
250
Distance to Union Carbide (km)

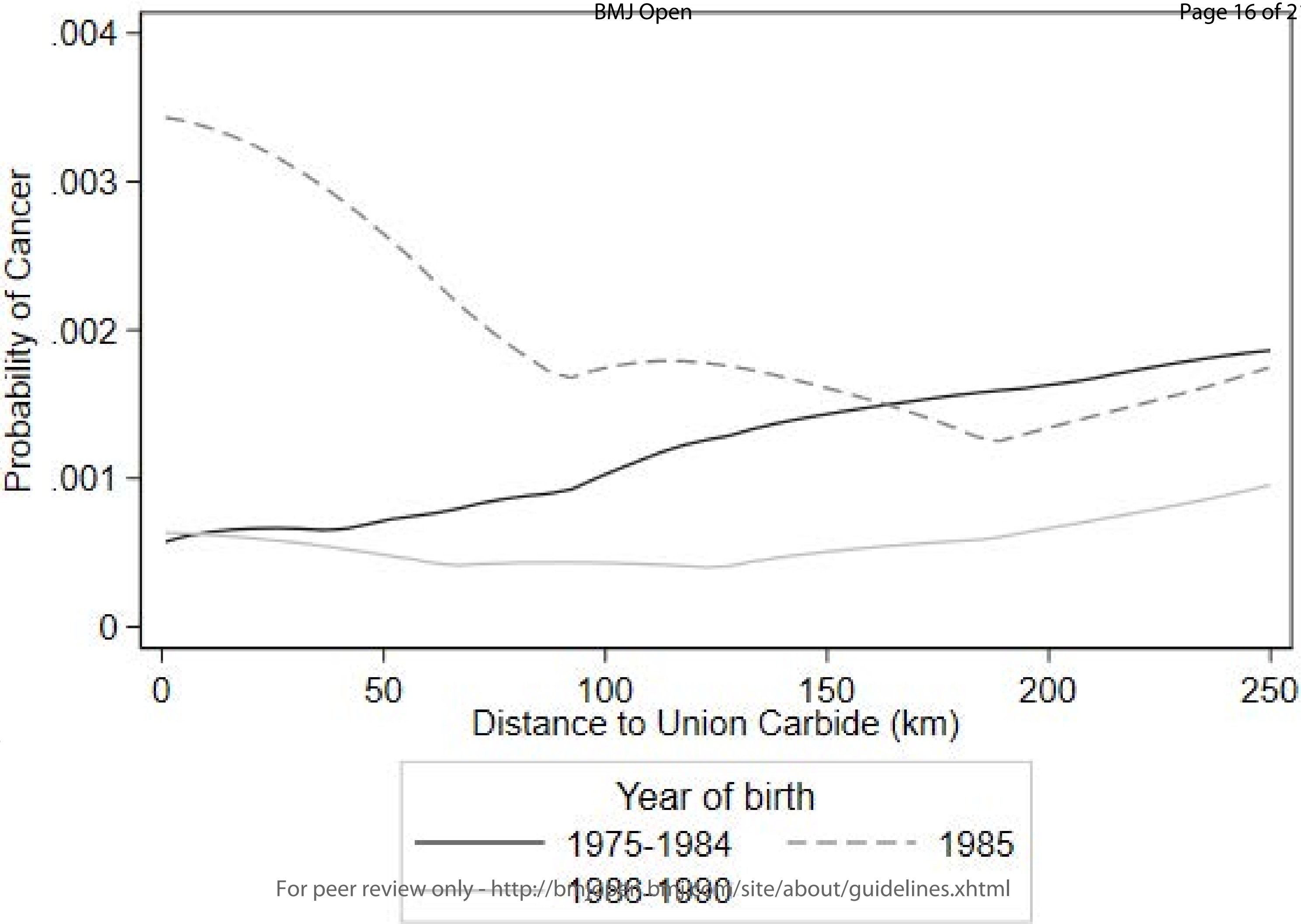

For peer review only - http://bmjopen.bmj.com/site/about/guidelines.xhtml

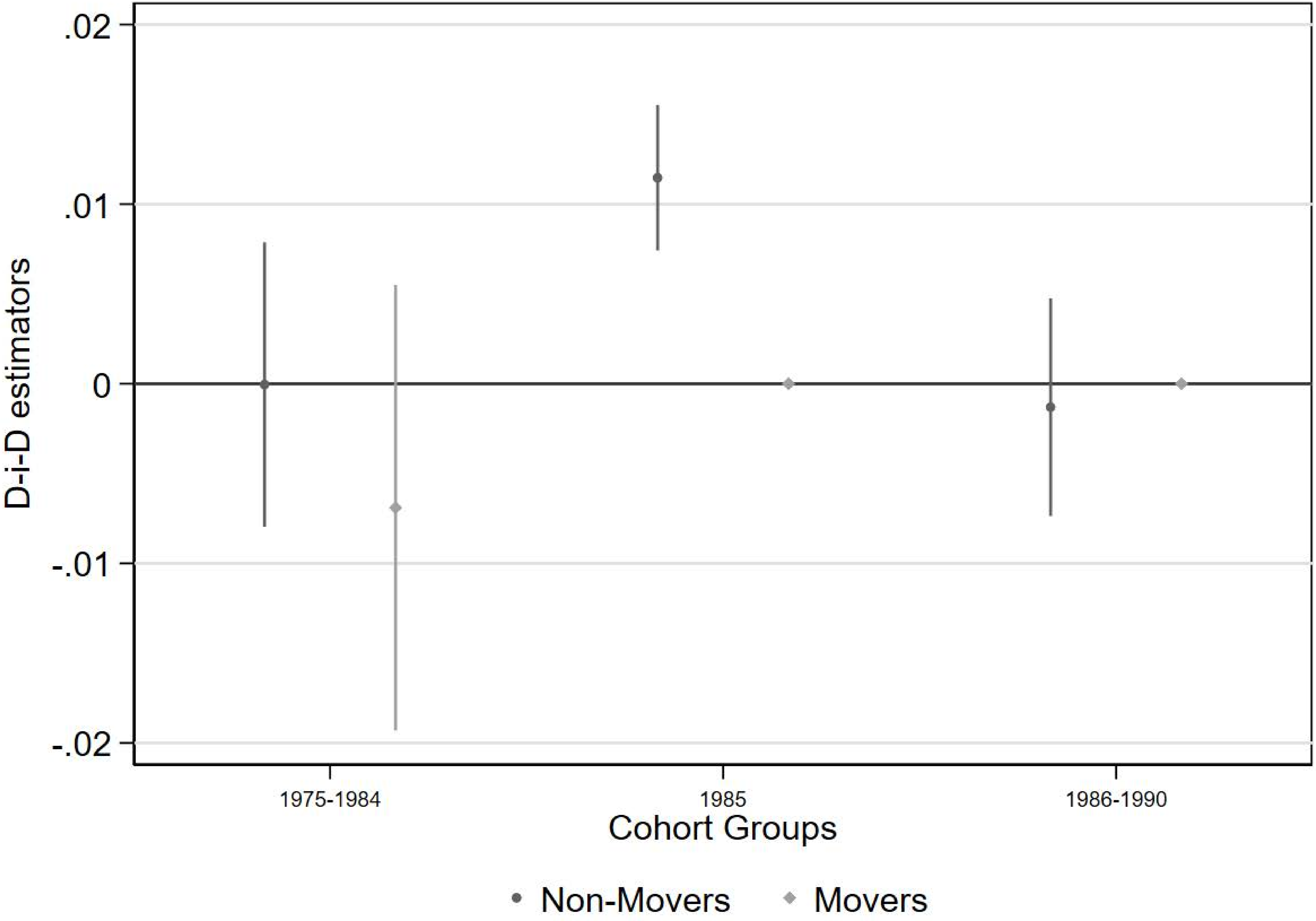
.02
.01
0
-.01
-.02
D-i-D estimators
1975-1984
1985
1986-1990
Cohort Groups
Non-Movers
Movers

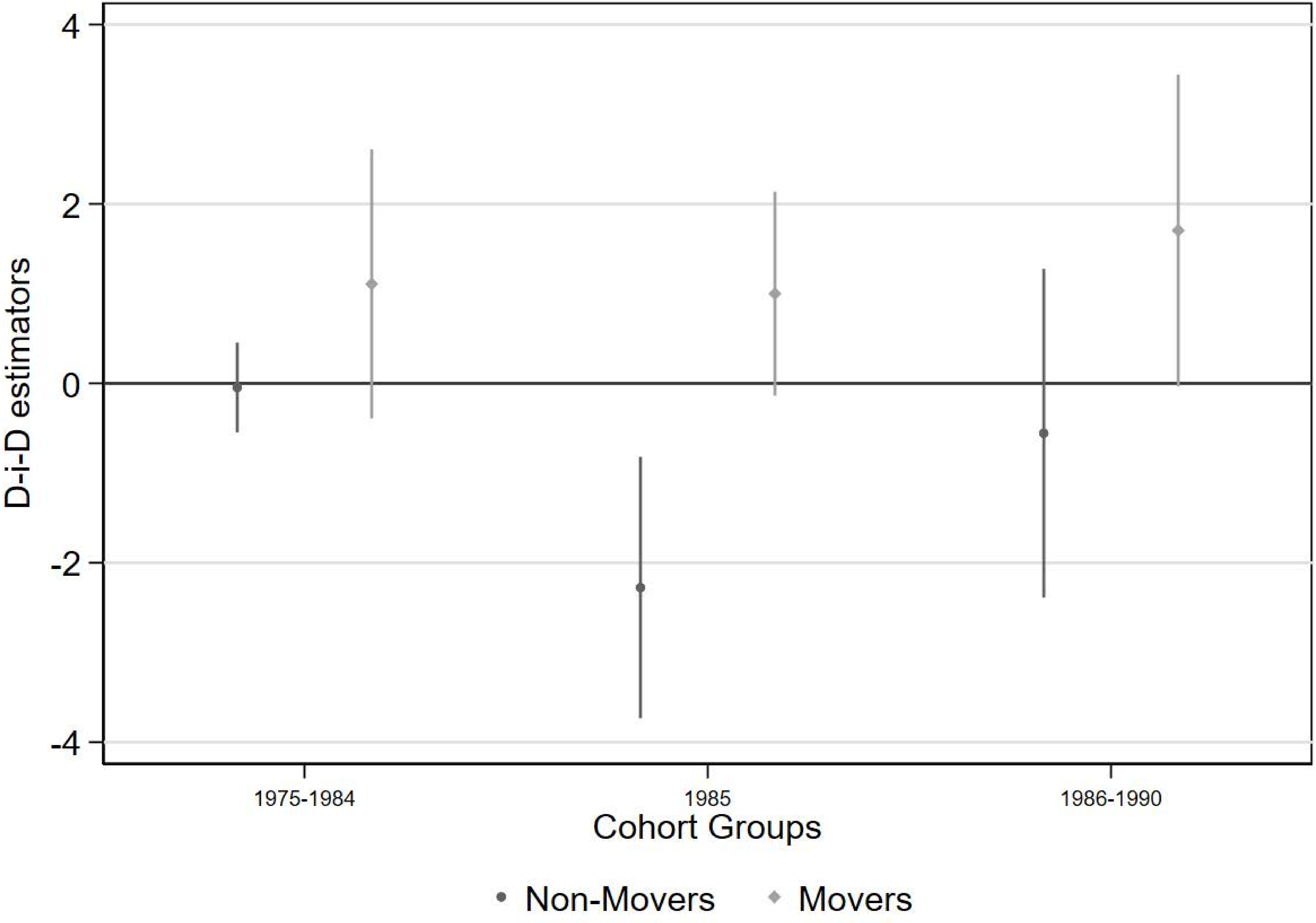
4
2
0
-2
-4
D-i-D estimators
1975-1984
1985
1986-1990
Cohort Groups
Non-Movers
Movers

In Utero Exposure to Industrial Disasters: A Case Study of the Bhopal Gas Tragedy

Supplemental Material

Gordon C. McCord[1]

Prashant Bharadwaj[2]

Lotus McDougal[3]

Arushi Kaushik[4]

Anita Raj[5]

Figure S1 uses the Indian Human Development Survey (IHDS) in a robustness check to test whether mothers of those who were in-utero at the time of the BGD are different from those of other cohorts. The IHDS-I was conducted in 2004-05 and is representative at the district level. It is the only other survey apart from the DHS that collects health information for India. We compare mothers' education level and several (self-reported) health parameters, such as the probability of reporting to be suffering from a heart disease, high blood pressure, diabetes, asthma, tuberculosis, leprosy, cancer and mental illness (n=1,792).

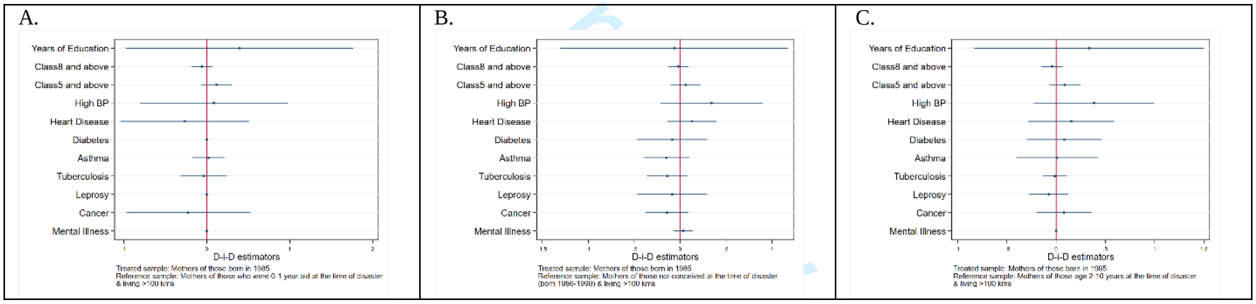

Figure S1: Characteristics of mothers of in-utero cohort compared to others

The figure shows difference-in-difference estimates for various outcome variables for the mothers of those who were in-utero (i.e, born in 1985). These estimates are obtained from regression equation similar to eq 1 where the control group includes mothers of non-mover men who were born between 1984 (i.e, < 1 year old at the time of BGD; panel A), non-mover men who were born between 1975-1983 (i.e, 2-10 years old at the time of BGD; panel B) and non-mover men who were born between 1986-1990 (i.e, not conceived at the time of BGD; panel C) and are living > 100kms away from Union Carbide plant. The figures depict estimates with 95% CI.

[1] Corresponding author. gmccord@ucsd.edu, School of Global Policy and Strategy, UC San Diego
[2] Department of Economics, UC San Diego
[3] Center on Gender Equity and Health, Department of Medicine, UC San Diego
[4] Department of Economics, UC San Diego
[5] Center on Gender Equity and Health, Department of Medicine, UC San Diego

For peer review only - http://bmjopen.bmj.com/site/about/guidelines.xhtml

Table S1: Linear probability models (LPM) for employment disability among men living in Madhya Pradesh in 1999 within 100km of Bhopal, compared to men of the same cohort more than 100km from Bhopal.

	Prob(Reported Employment Disability)		
	(1)	(2)	(3)
	Pooled	Non-Movers	Movers
1986-1990	-0.00109	-0.00131	0
	(0.00257)	(0.00297)	(5.63e-11)
1985	0.0111***	0.0115***	0
	(0.00216)	(0.00198)	(9.65e-11)
1975-1984	-0.000632	-4.65e-05	-0.00690
	(0.00344)	(0.00388)	(0.00608)
1960-1974	Reference	Reference	Reference
Observations	13,369	12,129	1,240
R-squared	0.003	0.003	0.024
Cohort FEs	Yes	Yes	Yes
Control Mean	0.00377	0.00426	0

Robust standard errors in parentheses
*** p<0.01, ** p<0.05, * p<0.1

Table S2: Regression results on educational attainment among men born in Madhya Pradesh within 100km of Bhopal, compared to men of the same cohort more than 100km from Bhopal.

	Years of Education Completed		
	(1)	(2)	(3)
	Pooled	Non-Movers	Movers
1986-1990	0.221	-0.557	1.704*
	(0.601)	(0.897)	(0.851)
1985	-0.577	-2.276***	0.999*
	(0.376)	(0.714)	(0.557)
1975-1984	0.255	-0.0471	1.108
	(0.279)	(0.245)	(0.734)
1960-1974	Reference	Reference	Reference
Observations	7,045	5,014	2,031
R-squared	0.048	0.055	0.047
Cohort FEs	Yes	Yes	Yes
Control Mean	5.833	5.561	6.600

Robust standard errors in parentheses
*** p<0.01, ** p<0.05, * p<0.1

For peer review only

For peer review only - http://bmjopen.bmj.com/site/about/guidelines.xhtml

STROBE Statement—Checklist of items that should be included in reports of ***cohort studies***

	Item No	Recommendation	Page No
**Title and abstract**	1	(*a*) Indicate the study's design with a commonly used term in the title or the abstract	1
		(*b*) Provide in the abstract an informative and balanced summary of what was done and what was found	1
**Introduction**			
Background/rationale	2	Explain the scientific background and rationale for the investigation being reported	2
Objectives	3	State specific objectives, including any prespecified hypotheses	3
**Methods**			
Study design	4	Present key elements of study design early in the paper	4
Setting	5	Describe the setting, locations, and relevant dates, including periods of recruitment, exposure, follow-up, and data collection	2-3
Participants	6	(*a*) Give the eligibility criteria, and the sources and methods of selection of participants. Describe methods of follow-up<br>(*b*) For matched studies, give matching criteria and number of exposed and unexposed	3
Variables	7	Clearly define all outcomes, exposures, predictors, potential confounders, and effect modifiers. Give diagnostic criteria, if applicable	3-4
Data sources/ measurement	8*	For each variable of interest, give sources of data and details of methods of assessment (measurement). Describe comparability of assessment methods if there is more than one group	3
Bias	9	Describe any efforts to address potential sources of bias	10
Study size	10	Explain how the study size was arrived at	3
Quantitative variables	11	Explain how quantitative variables were handled in the analyses. If applicable, describe which groupings were chosen and why	3
Statistical methods	12	(*a*) Describe all statistical methods, including those used to control for confounding<br>(*b*) Describe any methods used to examine subgroups and interactions<br>(*c*) Explain how missing data were addressed<br>(*d*) If applicable, explain how loss to follow-up was addressed<br>(*e*) Describe any sensitivity analyses	5
**Results**			
Participants	13*	(a) Report numbers of individuals at each stage of study—eg numbers potentially eligible, examined for eligibility, confirmed eligible, included in the study, completing follow-up, and analysed<br>(b) Give reasons for non-participation at each stage<br>(c) Consider use of a flow diagram	3
Descriptive data	14*	(a) Give characteristics of study participants (eg demographic, clinical, social) and information on exposures and potential confounders<br>(b) Indicate number of participants with missing data for each variable of interest<br>(c) Summarise follow-up time (eg, average and total amount)	3
Outcome data	15*	Report numbers of outcome events or summary measures over time	4

For peer review only

For peer review only - http://bmjopen.bmj.com/site/about/guidelines.xhtml

Main results	16	(*a*) Give unadjusted estimates and, if applicable, confounder-adjusted estimates and their precision (eg, 95% confidence interval). Make clear which confounders were adjusted for and why they were included<br>(*b*) Report category boundaries when continuous variables were categorized<br>(*c*) If relevant, consider translating estimates of relative risk into absolute risk for a meaningful time period	7
Other analyses	17	Report other analyses done—eg analyses of subgroups and interactions, and sensitivity analyses	7-9
**Discussion**			
Key results	18	Summarise key results with reference to study objectives	9
Limitations	19	Discuss limitations of the study, taking into account sources of potential bias or imprecision. Discuss both direction and magnitude of any potential bias	10
Interpretation	20	Give a cautious overall interpretation of results considering objectives, limitations, multiplicity of analyses, results from similar studies, and other relevant evidence	10
Generalisability	21	Discuss the generalisability (external validity) of the study results	10
**Other information**			
Funding	22	Give the source of funding and the role of the funders for the present study and, if applicable, for the original study on which the present article is based	1

*Give information separately for exposed and unexposed groups.

Note: An Explanation and Elaboration article discusses each checklist item and gives methodological background and published examples of transparent reporting. The STROBE checklist is best used in conjunction with this article (freely available on the Web sites of PLoS Medicine at http://www.plosmedicine.org/, Annals of Internal Medicine at http://www.annals.org/, and Epidemiology at http://www.epidem.com/). Information on the STROBE Initiative is available at http://www.strobe-statement.org.

For peer review only

BMJ Open

In Utero Exposure to Industrial Disasters: A Case Study of the Bhopal Gas Tragedy

Journal:	*BMJ Open*
Manuscript ID	bmjopen-2022-066733.R1
Article Type:	Original research
Date Submitted by the Author:	23-Feb-2023
Complete List of Authors:	McCord, Gordon C ; University of California San Diego, School of Global Policy and Strategy<br>Bharadwaj, Prashant; University of California San Diego, Department of Economics<br>McDougal, Lotus; University of California San Diego, Department of Medicine<br>Kaushik, Arushi; University of California San Diego, Department of Economics<br>Raj, Anita; University of California San Diego, Department of Medicine
**Primary Subject Heading**:	Public health
Secondary Subject Heading:	Global health
Keywords:	PUBLIC HEALTH, EPIDEMIOLOGY, HEALTH ECONOMICS

SCHOLARONE™
Manuscripts

I, the Submitting Author has the right to grant and does grant on behalf of all authors of the Work (as defined in the below author licence), an exclusive licence and/or a non-exclusive licence for contributions from authors who are: i) UK Crown employees; ii) where BMJ has agreed a CC-BY licence shall apply, and/or iii) in accordance with the terms applicable for US Federal Government officers or employees acting as part of their official duties; on a worldwide, perpetual, irrevocable, royalty-free basis to BMJ Publishing Group Ltd ("BMJ") its licensees and where the relevant Journal is co-owned by BMJ to the co-owners of the Journal, to publish the Work in this journal and any other BMJ products and to exploit all rights, as set out in our licence.

The Submitting Author accepts and understands that any supply made under these terms is made by BMJ to the Submitting Author unless you are acting as an employee on behalf of your employer or a postgraduate student of an affiliated institution which is paying any applicable article publishing charge ("APC") for Open Access articles. Where the Submitting Author wishes to make the Work available on an Open Access basis (and intends to pay the relevant APC), the terms of reuse of such Open Access shall be governed by a Creative Commons licence – details of these licences and which Creative Commons licence will apply to this Work are set out in our licence referred to above.

Other than as permitted in any relevant BMJ Author's Self Archiving Policies, I confirm this Work has not been accepted for publication elsewhere, is not being considered for publication elsewhere and does not duplicate material already published. I confirm all authors consent to publication of this Work and authorise the granting of this licence.

For peer review only

In Utero Exposure to Industrial Disasters: A Case Study of the Bhopal Gas Tragedy

Gordon C. McCord[1]

Prashant Bharadwaj[2]

Lotus McDougal[3]

Arushi Kaushik[4]

Anita Raj[5]

[1] Corresponding author. gmccord@ucsd.edu, School of Global Policy and Strategy, UC San Diego
[2] Department of Economics, UC San Diego
[3] Center on Gender Equity and Health, Department of Medicine, UC San Diego
[4] Department of Economics, UC San Diego
[5] Center on Gender Equity and Health, Department of Medicine, UC San Diego

Abstract

Objectives: Globalization and industrialization can increase economic opportunity for low- and middle-income nations, but these processes may also increase industrial accidents and harm workers. This paper examines the long-term, cohort-based health effects of the Bhopal Gas Disaster (BGD), one of the most serious industrial accidents in history.
Design: This retrospective analysis uses geolocated data on health and education from India's National Family Health Survey-4 (NFHS-4) and the 1999 Indian Socio-Economic Survey [NSSO-1999] to examine the health effects of exposure to the BGD among men and women aged 15-49 living in Madhya Pradesh in 2015-16 (women n=40,786; men n=7,031 [NFHS-4] and n=13,369 [NSSO-1999]), as well as their children (n=1,260). A spatial difference-in-differences strategy estimated the relative effect of being in utero near Bhopal relative to other cohorts and to those further from Bhopal separately for each dataset.
Results: We document long-term, intergenerational impacts of the BGD, showing that men who were in utero at the time were more likely to have a disability that affected their employment 15 years later, and had higher rates of cancer and lower educational attainment over 30 years later. Changes in the sex ratio among children born in 1985 suggest an effect of the BGD up to 100km from the accident.
Conclusions: These results indicate social costs stemming from the BGD that extend far beyond the mortality and morbidity experienced in the immediate aftermath. Quantifying these multigenerational impacts are important for policy consideration. Moreover, our results suggest that the BGD affected people across a substantially more widespread area than has previously been demonstrated.

Ethical approval: All data used in this study are publicly available and de-identified. Ethical approval for NFHS-4 data collection was provided by the International Institute for Population Sciences and ICF. Ethical approval for the 1999 NSSO Socio-Economic Survey was provided by the Indian National Sample Survey Office. Ethical exemption for this analysis was provided by the University of California San Diego IRB.

Transparency statement: The authors affirm that the manuscript is an honest, accurate, and transparent account of the study being reported; that no important aspects of the study have been omitted.

For peer review only - http://bmjopen.bmj.com/site/about/guidelines.xhtml

Funding: AR and LM received funding from the Bill and Melinda Gates Foundation (INV-008648).

Strengths and Limitations of this Study

- This study is the first, to our knowledge, to use different birth cohorts and a geographic difference-in-difference strategy to attribute causality of subsequent health and social consequences to the Bhopal Gas Disaster.
- The design nets out any difference in outcomes that arise due to time invariant aspects of being close to Bhopal, as well as aspects of belonging to a specific birth cohort.
- As with any ecological exposure assignment, the cohort of in utero children we assign to exposure will include a range of actual exposure to MIC gas.
- Mortality can affect the composition of the population observed post-facto; if the weakest children in utero were more likely to die from the incident, then our estimates could be considered a lower bound.
- The long-term consequences that we estimate could be the result of both direct effects from exposure as well as lack of subsequent mitigation of the effects through health, disability and education services.

Introduction

Globalization and industrialization can increase economic opportunity for low- and middle-income nations, but recent cross-national evidence indicates that these processes may also be increasing industrial accidents and harming the workers of these nations.[1] Evidence from India suggests high numbers of industrial accidents, with the highest associated attributable deaths in the world.[2 3] Despite recommendations for improved protections for workers and industry in India,[3] the Ministry of Environment, Forest and Climate Change has proposed the Environmental Impact Assessment (EIA) notification 2020, which will roll back environmental and worker protections in the country. Critics, including United Nations experts, have stated that this rollback violates the safety and health of workers and the environment and with potential impact on subsequent generations.[4-6] Unfortunately, little research is available on these multigenerational impacts, and inadequate recognition of these far-reaching repercussions can diminish the memory of such tragedies for policy consideration. This research provides insight into these impacts on children born to women survivors of one of the worst industrial disasters in history, the Bhopal Gas Disaster.

The Bhopal Gas Disaster (BGD) occurred in December 1984 and involved a methyl isocyanate (MIC) gas leak at a Union Carbide pesticide plant near the city of Bhopal, in Madhya Pradesh, India. The leak spread toxic gas in approximately a 7 km radius around the plant, exposing more than half a million people and resulting in up to 30,000 deaths in the region.[7] There is a broad spectrum of serious long-term and chronic health consequences for hundreds of thousands of survivors, including children, manifesting across multiple systems (e.g. respiratory, neurologic, musculoskeletal, ophthalmic, endocrine).[7-10] These impacts may be the tip of the iceberg however, given that MIC toxins affected groundwater[11] and the reproductive health and other health outcomes of exposed women,[8] factors suggesting that generations not exposed to the toxic gas directly may nevertheless suffer adverse health and social impacts of the BGD event. Potential in utero effects cannot be understated. Studies document a 4-fold increase in the rate of miscarriage following the gas leak, as well as increased risk for stillbirth and neonatal mortality.[9 10] Decades after the disaster, menstrual abnormalities and premature menopause have emerged as common

For peer review only - http://bmjopen.bmj.com/site/about/guidelines.xhtml

problems among exposed women and their female offspring.[9] MIC has also been shown to damage human chromosomes.[12] Early clinical studies on the gas-affected population showed signs of increased chromosomal aberrations that could manifest as cancer,[13] though population-level changes in cancer rates 8 years after the accident were suggestive but not significant.[14]

This paper estimates the long-term health (specifically, adult cancer rates and disability) and human capital impacts (educational attainment) on individuals who were exposed to the BGD in utero or as children in 1984. Understanding the long-term impacts of industrial disasters on children, especially those in utero at the time of the disaster, is important for several key reasons. First, while the immediate damage to children in the form of direct health impacts is perhaps easier to assess, the long-term, health-related damages could take years to manifest and might not be part of the legal damages considered. Second, economic research over the last few decades has shown that interventions that improve outcomes for children have high rates of return;[15] to this second point, we include education as an important indicator of socioeconomic impact on these children, particularly as these secondary effects may compound health impacts. Our findings offer timely information to inform debate on potential rollbacks of policies related to social and environmental protections attached to industrialization in India and elsewhere.

Methods

Data

The principal data source is the fourth round of the Demographic and Health Surveys (DHS) in India, which was conducted between 2015-2016 (also called National Family Health Survey, or NFHS-4, in India).[16] The DHS/NFHS-4 is a high-quality household survey that provides detailed information on health at the individual level, and is representative at both the national and state levels. All interviews in Madhya Pradesh were completed in 2015. For the first analysis focusing on differences in the sex ratio, the analytic sample includes children born between 1981-1985 to women who were interviewed in Madhya Pradesh and who lived within 250 km of Bhopal (n=1,260 births). The analytic sample for the analysis of cancer incidence includes men and women who were interviewed in Madhya Pradesh and were born between 1960 and 1990 (n=7,031 men and 40,786 women).

A second dataset that we use is the Integrated Public Use Microdata Series (IPUMS) from India for the year 1999, which presents harmonized data from the 1999 Socio-Economic Survey conducted by the National Sample Survey Organization (NSSO).[17] These data contain employment information for a representative sample, but only identifies the district of the respondent. This is the most recent NSSO survey in which respondents were asked how long they had been living at their current residence, a crucial question for this analysis. The survey sample includes people from ages 6-64, thus including those who were in utero at the time of the BGD. The analytic sample for this analysis includes men interviewed in Madhya Pradesh and born between 1960 and 1990 (n=13,369)

These two datasets could not be merged at the individual level, as the same respondents were not interviewed in each survey. In order to preserve the more precise geolocation of the NHFS-4, the two datasets were analyzed separately.

Patient and Public Involvement

Since DHS data are anonymized, direct involvement with study subjects is not possible. Once published, results will be disseminated to the public in Bhopal through a letter to local newspaper media.

For peer review only - http://bmjopen.bmj.com/site/about/guidelines.xhtml

Measures

For the analysis of children born to women surveyed by the NFHS-4, the outcome of interest was the ratio of male-to-female births.

For men, the outcomes of interest were cancer rates and educational attainment (from the NFHS-4) and employment disability (from the NSSO). Cancer was assessed as a general question "Do you currently have cancer", and educational attainment was assessed as the number of years that an individual attended school. Of the sample of men, 17 out of 7,031 with nonmissing response reported to have cancer, and the average number of years of education was 7.0 with a standard deviation of 5.1. We use the question on whether "the respondent was economically inactive because of disabilities or...other health-related reasons" to classify the person as suffering from employment disability. Forty-six men in the sample of 13,369 report to be suffering from employment disability. Given the prevalence of child labor, note that the employment-related questions of the survey are asked of all respondents, including children. Analyses assessing *movers* vs. *non-movers* are necessarily restricted to men, since patrilocality in India means that most women move to their husband's house after marriage.[18] As 78% of Indian women aged 15-49 have married,[16] the sample size of non-mover females is both small and non-representative, and thus cannot be considered in this analysis.

For all analyses, distance from the Union Carbide plant was the primary predictor. The NFHS-4 collected the latitude and longitude of each respondent's sampling cluster (henceforth, DHS clusters). The DHS clusters are villages or hamlets in rural areas and blocks or census tracts in urban areas; thus, they are much smaller geographical units than administrative units such as districts. These coordinates enable the calculation of distance from the Union Carbide plant in Bhopal for each respondent, within a privacy displacement radius of 2 km (urban) or 5-10 km (rural) (Figure 1).

Figure 1: Madhya Pradesh and DHS cluster locations around Bhopal

In the case of the district-level NSSO data, we measure distance from the Union Carbide plant to the nearest border of the district. For both datasets, we use the distance from the plant to form the treatment and control groups, since exposure to the toxic MIC gas would be more muted at greater distances.

Analysis

The analysis uses a spatial difference-in-differences strategy, where distance from the Union Carbide plant (we also refer to this as "distance from Bhopal") lends the spatial dimension. We compare people living close to Bhopal to those far away from Bhopal, and people who were in utero at the time of the BGD to those who were older or not yet conceived at the time of BGD. Under some standard assumptions,[19] these estimates net out any difference in outcomes that arise due to *time invariant* aspects of being close to Bhopal, as well as aspects of belonging to a specific birth cohort. The estimation also nets out the effect of other programs that might have been occurring during these years across multiple birth cohorts, such as the Universal Immunization Program.

All analyses were conducted using birth year cohorts (Figure 2). The reference cohort includes those born between 1960-1974 (i.e. respondents who were more than 10 years old at the time of the BGD). Three other cohorts are considered: young children below 10 years old at the time of the BGD (i.e., respondents born between 1975-1984), fetuses (i.e. respondents born in 1985) and children not yet conceived at the time of BGD (i.e. respondents born between 1986-1990). This last cohort can be considered a placebo

For peer review only - http://bmjopen.bmj.com/site/about/guidelines.xhtml

test, as we expect effects of the BGD on children not yet conceived to be null or significantly smaller than for cohorts that were alive or in utero.

Figure 2: Description of all cohorts used in empirical estimations.

It is worth noting that all four cohorts have been affected by the disaster, including our reference cohort. Therefore, while older cohorts are being used as a reference category, our results speak to the *relative* impacts on those who were in utero at the time of the disaster.

Linear regression models were used to estimate the relationship between proximity to Bhopal, birth cohort, and outcomes of interest, with the basic specification as follows:

$$\text{(I)} \qquad Y_{itc} = \alpha_1 Bhopal_c + \beta_1 * T1_t * Bhopal_c + \beta_2 * T2_t * Bhopal_c + \beta_3 * T3_t * Bhopal_c + \text{Cohort FE} + \varepsilon_{itc}$$

where Y_{itc} is the outcome of interest for person *i* born in year *t* and living in location *c*. We consider three health and education outcomes, namely: the probability of suffering from cancer, the probability of suffering from employment disability, and years of education completed. $Bhopal_c$ takes the value of 1 if the respondent *i* is living near Bhopal (we define "near" Bhopal as being within 100kms following spatial patterns in sex ratios explained in Figure 3) and 0 otherwise. $T1_t$ is a dummy variable equal to 1 if the respondent was born between 1975-1984 (cohort of ages 0-10 at the time of BGD) and 0 otherwise. $T2_t$ is a dummy variable equal to 1 if the respondent was born in 1985 (in utero cohort) and 0 otherwise. $T3_t$ is a dummy variable equal to 1 if the respondent was born between 1986-1990 (cohort not yet conceived at the time of BGD) and 0 otherwise. The omitted category consists of respondents who were older than 10 years at the time of the disaster and are living more than 100 kms away from Bhopal. Cohort FE refers to birth year fixed effects to standardize the outcome variable across ages. Standard errors are two-way clustered by district and birth year in order to account for spatial and serial autocorrelation within district, as well as covariate shocks across all people in the same birth cohort (such as changes in food prices).

β_1, β_2, and β_3 respectively capture the effect of BGD on young children below 10 years old, fetuses and not-yet-conceived children relative to those who were older than 10 at the time of BGD. The spatial difference-in-difference compares children in a cohort living near Bhopal to children in the same cohort living far from Bhopal. In order to interpret β_1, β_2, and β_3 as the causal effect of the BGP, the underlying identifying assumption is that in the absence of the disaster the cohorts near and far from Bhopal would have evolved similarly. Note that α_1 captures the effect of living near Bhopal vis-a-vis far from Bhopal for the reference cohort (1965-1974). This difference captures time invariant differences in the two geographical locations.

Results

Sex ratio changes following the Bhopal Gas Disaster

The sex ratio of children born between 1981-1984 is markedly different from that of children born in 1985 (Figure 3). Women who lived within 100 km of Bhopal experienced a relative decrease in the birth of males in the 1985 cohort (64% of children born from 1981-1984 were male, a proportion that drops to 60% in 1985). This is consistent with male fetuses more affected by external stress.[20-22] Women living beyond 100km had no difference in the sex ratio across the 1981-1984 and the 1985 cohorts.

For peer review only - http://bmjopen.bmj.com/site/about/guidelines.xhtml

Figure 3. Difference in male-female sex ratio between 1985 and 1981-1984 cohorts among children born to women ages 15-49 in Madhya Pradesh, by distance to Union Carbide plant.

Adult Cancer Rates among Exposed Children

Similarly to patterns seen with changes in the sex ratio, children who were born in 1985 (the year following the BGD) and who lived close to Bhopal during the disaster experience much higher rates of cancer as adults compared to adults who were born before or after the disaster and who lived further away from Bhopal (Figure 4). As in the case of differences in sex ratios, the abnormally high cancer rates appear to extend up to 100 km from Bhopal. Given the two spatial patterns in Figures 3 & 4, we use 100 km as the BGD exposure cutoff in our analysis.

Figure 4. Self-reported cancer incidence among men and women living in Madhya Pradesh in 2015, by distance to Union Carbide plant.

Among all men in the NFHS-4 analytic sample, estimates suggest that the 1975-1984 and 1986-1990 birth cohorts have statistically indistinguishable cancer rates compared to the 1960-1974 cohort, while men born within 100 km of Bhopal in 1985 have a 2.1 percentage point higher risk than the other cohorts (Table 1). This is an 8-fold higher cancer risk compared to the other cohorts.

Table 1: Probability of suffering from cancer among men born in Madhya Pradesh.

	Prob(Reports having Cancer)		
	(1)	(2)	(3)
	Pooled	Non-Movers	Movers
1986-1990	-0.00373	0.00170	-0.0141
	(0.00340)	(0.00147)	(0.0121)
1985	0.0209***	0.0624***	-0.00906
	(0.00591)	(0.00505)	(0.0113)
1975-1984	-0.00459	-0.00157	-0.00906
	(0.00355)	(0.00181)	(0.0113)
1960-1974	Reference	Reference	Reference
Observations	7,031	5,002	2,029
R-squared	0.007	0.012	0.014
Cohort FEs	Yes	Yes	Yes
Control Mean	0.00252	0.00228	0.00321
Robust standard errors in parentheses			
*** p<0.01, ** p<0.05, * p<0.1			

Notes: The cohort of men born in 1985 within 100 km of Bhopal has a higher likelihood of cancer compared to other cohorts and to those born in Madhya Pradesh beyond 100 km from Bhopal (1), with effect most pronounced among men who have never moved (2) instead of those who have moved (3). Estimates are from ordinary least squares regressions as in Equation (I) with controls consisting of fixed effects for year of birth cohort and whether the DHS cluster is within 100 km of Bhopal. Standard errors are two-way clustered at the district and year-of-birth levels.

For peer review only - http://bmjopen.bmj.com/site/about/guidelines.xhtml

Among men who have never moved, and were therefore residing within 100km of the Union Carbide plant during the BGD, the effect of being born in 1985 and near Bhopal is even stronger. These men have a 6.2 percentage point higher cancer risk in 2015 compared to the other cohorts and to those living more than 100 km from Bhopal.[1] This represents a 27-fold higher risk of cancer among adults who were in utero during the BGD, suggesting long-term health consequence of exposure, even while in the womb. Men who moved at some point before the survey in 2015/16 (and who were therefore less likely to be exposed to the BGD), and had no difference in cancer rates across cohorts.

Effects on Employment Disability

Men who were in utero during the BGD and who lived in districts with a border within 100 km Bhopal are 1 percentage point more likely to report employment disability when they are surveyed as adults, compared to the older cohorts and those living further from Bhopal (Figure 5; Table S1). The effect increases to 2 percentage points among those living within 50 km of Bhopal (Table S2), consistent with higher intensity of exposure to MIC among those living closer to Bhopal.

Figure 5: Employment disability, compared to reference group of men born from 1960-1974 and living > 100 km from Bhopal. Bars represent 95% confidence intervals. Note that there were no individuals reporting employment disability in the 1985 and 1986-1990 cohorts among the *movers* subgroup.

Effects on Educational Attainment

Relative to other cohorts, men who were in utero during the BGD attained approximately two fewer years of education, with the effects only evident among men who have never moved (Figure 6; Table S3). Limiting the treatment radius to 75 km yields the same estimated effect, while smaller distances yield no discernible effect (Table S4).

Figure 6: Years of education completed, compared to reference group of men born from 1960-1974 and living > 100 km from Bhopal.

Discussion

The Bhopal Gas Disaster was one the most serious industrial accidents in history, causing thousands of deaths at the time of the leak, and tens of thousands more deaths and serious health sequelae in the three decades since.[23 24] This analysis documents long-term, intergenerational impacts of the BGD, showing that men who were in utero at the time of the BGD were more likely to have a disability that affected their employment 15 years later, and had higher rates of cancer and lower educational attainment over 30 years later. Importantly, these results indicate social costs stemming from the BGD that extend far beyond the mortality and morbidity experienced in the immediate aftermath. Moreover, our results suggest that the BGD affected people across a substantially more widespread area than has previously been demonstrated:

[1] Given the presence of both fixed effects and interaction terms in the difference-in-differences framework, the linear probability model estimated with ordinary least squares is more appropriate than logistic regression. For completeness, we report the odds ratio for the 1985 cohort from a conditional logic model: 3.36 (0.1-111.8). While the estimation problems with logistic in the difference-in-differences context means we cannot interpret the magnitude, the sign is consistent with the results we report from ordinary least squares.

For peer review only - http://bmjopen.bmj.com/site/about/guidelines.xhtml

up to around 100 km (approximately 60 miles) from the site, as opposed to the 4.5 km radius that was considered exposed by public health officials and researchers after the disaster.[9 10 14 25] This study is also the first, to our knowledge, to use different birth cohorts to attribute causality of subsequent health and social consequences to the BGD.

We find that men currently living within 100 km of Bhopal and born in 1985 have an 8-fold higher risk of cancer than men of other birth cohorts; of those men, those who have never changed residence since the BGD have a 27-fold higher risk of cancer. While elevated cancer levels have been previously associated with MIC exposure,[14 26-28] our results identify long-term, birth-year based differences in cancer prevalence. This underscores the plausibility of multiple MIC exposure pathways, including teratogenic in utero exposure, and ongoing environmental exposure. Bhopal has ongoing cancer surveillance programs,[29] and it remains clear that those exposed to MIC require ongoing and careful monitoring, support and treatment.

Employment disability was one percentage point more likely among men who were in utero in districts within 100 km of Bhopal during the BGD than those born prior or more distal to the BGD. This is a meaningful impact since baseline employment disability rates are quite low (0.4%), and men's employment at the time of study was nearly universal at 98%.[16] In a context of high poverty, with nearly 40% of Madhya Pradesh's population living beneath the poverty line,[30] more than 90% informal employment nationally (thus lacking social protections and safety nets),[31] and household reliance on male income due to low levels of female labor force participation and a highly skewed gender wage gap,[16 32] this increase in disability or health-related economic inactivity risks substantial impact on the health, safety and well-being of affected families.

The finding that men who were in utero and within 100km of Bhopal during the BGD received more than two fewer years of education than other cohorts has tremendous human capital implications. This is a large impact both since the average number of years of education in the control group is only 5.6 years, and because education has such a direct association with subsequent wages and consumption.[33] We cannot ascertain whether the lower educational attainment is due to health or cognitive consequences of being exposed to the BGD in utero. Given that the BGD effect is much more evident in the 1985 cohort compared to older or younger children, it is unlikely that the effect operated through broader socioeconomic impacts or impacts on other family members that limited children's access to school. Regardless of the mechanism, the reduced educational attainment is evidence of long-term consequences of exposure to the accident that imply personal and social costs far beyond the immediate health impacts.

A few study limitations are worth outlining. First, as with any ecological exposure assignment, the cohort of in utero children we assign to exposure will include a range of actual exposure to MIC gas. The interpretation of our estimates would be the effect of average exposure in the population we define as treated. Secondly, our estimates are affected by mortality and migration. An important consequence of a disaster, whether natural or man-made, is that mortality can severely affect the composition of the population observed post-facto. In our empirical context, any mortality effect of the disaster that symmetrically affects all cohorts within Bhopal (without affecting people away from Bhopal) will not affect our estimates. As such, it is differential mortality across cohorts due to the BGD that would have compositional effects on our study sample. For example, if the weakest children in utero were more likely to die from the incident, then our estimates could be considered a lower bound.

Likewise, a frequent human response to a disaster is migration to safer areas. Again, it would only be differential migration in response to the BGD (different migration across cohorts and across people living close or far from Bhopal) that would create a problematic compositional difference between our treated cohort and the others. In fact, migration in this area was relatively low and researchers found that 91% of the population remained in the same area subsequent to 1984.[10] Our own estimates in Figure S1 suggest

For peer review only - http://bmjopen.bmj.com/site/about/guidelines.xhtml

that mothers of the in utero cohort are not significantly different from mothers of other consecutive cohorts on various educational and health dimensions.

It is also important to note that the long-term consequences that we estimate could be the result of both direct effects from exposure as well as lack of subsequent mitigation of the effects through health, disability and education services. While disentangling these forces is important from a policy perspective, in this paper we simply highlight the total combined effect of being exposed to such disasters as a child. As such, causal inference methods mitigate but do not eliminate the need to consider different routes of causation.

Work in other empirical contexts of industrial disasters should strive to estimate how effects on in utero children vary by trimester of pregnancy. Other kinds of shocks have been shown to vary in their effect by trimester,[34] however given data quality issues on long recall of month-of-birth in the DHS,[35] we do not attempt to estimate effects by trimester in this study.

Finally, cancer reporting in the data comes from self-reports, which could be subject to biases; there are also very few cases of self-reported cancer in the data. We think of our results on cancer as being consistent with the broader public health narrative on the consequences of the disaster, but more data through systematic cancer screening and place of birth information is key to firmly establish this link.

Understanding the short- and long-term damages caused by industrial disasters is key to gaining insight into the trade-offs involved in making regulatory decisions. It is also crucial from a policy response perspective if policy-makers wish to dedicate resources to mitigate harm done by such events or to compute legal damages. These concerns are particularly germane now, when there is evidence that the multiple health conditions suffered by many BGD survivors may make them more susceptible to the COVID-19 pandemic.[36 37] The evidence presented in this paper starkly highlights the long-term, intergenerational health and human capital effects of the BGD, and underscores the need for ongoing survivor support, as well as robust regulatory protections.

Funding Statement

AR and LM received funding from the Bill and Melinda Gates Foundation (INV-008648).

Declaration of interests

The authors declare no conflict of interest.

Author contributions

PB conceptualized the project, GCM and AK organized data and conducted analysis, all authors including LM and AR contributed equally to data interpretation, writing, review and editing. GCM and AK have verified the underlying data.

For peer review only - http://bmjopen.bmj.com/site/about/guidelines.xhtml

Ethics Statement

All data used in this study is publicly available and de-identified. Ethical approval for NFHS-4 data collection was provided by the International Institute for Population Sciences and ICF. Ethical approval for the 1999 NSSO Socio-Economic Survey was provided by the Indian National Sample Survey Office. Ethical exemption for this analysis was provided by the University of California San Diego IRB.

Data Sharing Statement

Replication code will be made available upon publication. The raw DHS survey data are subject to a user agreement and are available at available from the Demographics and Health Surveys Program at https://dhsprogram.com. The IPUMS data with the 1999 National Sample Survey are available from https://international.ipums.org/international/index.shtml.

The Corresponding Author has the right to grant on behalf of all authors and does grant on behalf of all authors, an exclusive licence (or non exclusive for government employees) on a worldwide basis to the BMJ Publishing Group Ltd to permit this article (if accepted) to be published in BMJ editions and any other BMJPGL products and sublicences such use and exploit all subsidiary rights, as set out in our licence.

For peer review only

For peer review only - http://bmjopen.bmj.com/site/about/guidelines.xhtml

References

1. Blanton RG, Peksen D. Dying for globalization? The impact of economic globalization on industrial accidents. *Social Science Quarterly* 2017;98(5):1487-502.
2. Nalapat S. Workplace disasters during lockdown put spotlight on India's patchy safety record -- explained with graphs. *Times Now News* 2020 September 7, 2020.
3. Sengupta A, Bandyopadhyay D, Van Westen C, et al. An evaluation of risk assessment framework for industrial accidents in India. *Journal of loss prevention in the process industries* 2016;41:295-302.
4. Explained: What is EIA 2020? How does it water down the existing policy? *The Week* 2020 August 10, 2020.
5. Explained Ideas: What ails with the draft EIA notification 2020? *The Indian Express* 2020 August 12, 2020.
6. UN experts to Govt of India: Environmental exemptions to industry unwarranted. 2020 September 4, 2020.
7. Sharma DC. Bhopal: 20 years on. *Lancet* 2005;365(9454):111-2. doi: 10.1016/S0140-6736(05)17722-8 [published Online First: 2005/01/12]
8. Ganguly BB, Mandal S, Kadam NN. Spectrum of health condition in methyl isocyanate (MIC)-exposed survivors measured after 30 years of disaster. *Environ Sci Pollut Res Int* 2018;25(5):4963-73. doi: 10.1007/s11356-017-0865-6 [published Online First: 2017/12/06]
9. Mishra PK, Samarth RM, Pathak N, et al. Bhopal Gas Tragedy: review of clinical and experimental findings after 25 years. *Int J Occup Med Environ Health* 2009;22(3):193-202. doi: 10.2478/v10001-009-0028-1 [published Online First: 2009/10/13]
10. Dhara VR, Dhara R. The Union Carbide disaster in Bhopal: a review of health effects. *Archives of Environmental Health: An International Journal* 2002;57(5):391-404.
11. Mishra B, Banerjee N. MIC leak disaster and environmental contamination: Time to act now. *Natl J Community Med* 2014;5:13-20.
12. Ennever FK, Rosenkranz HS. Evaluating the potential for genotoxic carcinogenicity of methyl isocyanate. *Toxicol Appl Pharmacol* 1987;91(3):502-05.
13. Malla T, Senthilkumar C, Sharma N, et al. Chromosome instability among Bhopal gas tragedy survivors. *Am Eur J Toxicol Sci* 2011;3:245-9.
14. Dikshit RP, Kanhere S. Cancer patterns of lung, oropharynx and oral cavity cancer in relation to gas exposure at Bhopal. *Cancer Causes Control* 1999;10(6):627-36.
15. Magnuson K, Duncan GJ. Can early childhood interventions decrease inequality of economic opportunity? *RSF: The Russell Sage Foundation Journal of the Social Sciences* 2016;2(2):123-41.

[dataset] 16. International Institute for Population Sciences (IIPS), ICF. National Family Health Survey (NFHS-4), 2015-16: India. Mumbai: IIPS, 2017.

[dataset] 17. National Sample Survey Office - M/o Statistics and Implementation (MOSPI), Government of India. India - Informal Non-Agricultural Enterprises Survey, July 1999 - June 2000, NSS 55th Round. In: NSSO, ed. Delhi, India, 1999-2000.

18. Khalil U, Mookerjee S. Patrilocal Residence and Women’s Social Status: Evidence from South Asia. *Economic Development and Cultural Change* 2019;67(2):401-38. doi: 10.1086/697584
19. Wooldridge J. Introductory Econometrics. 7th ed: Cengage Learning 2020.
20. Kraemer S. The fragile male. *BMJ (Clinical research ed)* 2000;321(7276):1609-12. doi: 10.1136/bmj.321.7276.1609
21. Hansen D, Moller H, Olsen J. Severe periconceptional life events and the sex ratio in offspring: follow up study based on five national registers. *BMJ (Clinical research ed)* 1999;319(7209):548-49. doi: 10.1136/bmj.319.7209.548
22. Mizuno R. The male/female ratio of fetal deaths and births in Japan. *Lancet* 2000;356(9231):738-9. doi: 10.1016/s0140-6736(00)02637-4

For peer review only - http://bmjopen.bmj.com/site/about/guidelines.xhtml

23. Broughton E. The Bhopal disaster and its aftermath: a review. *Environmental Health* 2005;4(1):6. doi: 10.1186/1476-069X-4-6
24. Odysseos L. The question concerning human rights and human rightlessness: disposability and struggle in the Bhopal gas disaster. *Third World Quarterly* 2015;36(6):1041-59.
25. Sathyamala C, Jayaprakash N. The Bhopal Disaster and Medical Research. *Economic and Political Weekly* 2012:37-40.
26. Khan I, Senthilkumar CS, Upadhyay N, et al. In silico docking of methyl isocyanate (MIC) and its hydrolytic product (1, 3-dimethylurea) shows significant interaction with DNA Methyltransferase 1 suggests cancer risk in Bhopal-Gas-Tragedy survivors. *Asian Pacific Journal of Cancer Prevention* 2015;16(17):7663-70.
27. Senthilkumar CS, Malla TM-u-D, Sah NK, et al. Cancer Morbidity Among Methyl Isocyanate Exposed Long-Term Survivors and Their Offspring: A Hospital-Based Five Year Descriptive Study (2006-2011) and Future Directions to Predict Cancer Risk in the Affected Population. *Asian Pacific journal of cancer prevention: APJCP* 2011;12(12):3443-52.
28. Senthilkumar CS, Akhter S, Malla TM, et al. Increased micronucleus frequency in peripheral blood lymphocytes contributes to cancer risk in the methyl isocyanate-affected population of Bhopal. *Asian Pac J Cancer Prev* 2015;16(10):4409-19. doi: 10.7314/apjcp.2015.16.10.4409 [published Online First: 2015/06/02]
29. National Centre for Disease Informatics and Research. Area Cancer National Cancer Registry Programme, India; 2021 [Available from: https://ncdirindia.org/Area_Cancer.aspx accessed June 8 2021.
30. Ministry of Social Justice and Empowerment, Department of Social Justice and Empowerment, Government of India. State-Wise Percentage of Population Below Poverty Line by Social Groups, 2004-05: Government of India; 2021 [Available from: http://socialjustice.nic.in/UserView/index?mid=76672 accessed June 8 2021.
31. Mehrotra S. Informal Employment Trends in the Indian Economy: Persistent informality, but growing positive development. In: Employment Policy Department I, ed. Employment: Working Paper No 254. Geneva, Switzerland: International Labour Organisation, 2019.
32. ILO Decent Work Team for South Asia and Country Office for India. India Wage Report: Wage policies for decent work and inclusive growth. Delhi, India: International Labour Organization, 2018.
33. Fulford S. Returns to education in India. *World Development* 2014;59:434-50.
34. Almond D, Mazumder B. Health Capital and the Prenatal Environment: The Effect of Ramadan Observance during Pregnancy. *American Economic Journal: Applied Economics* 2011;3(4):56-85. doi: 10.1257/app.3.4.56
35. Larsen AF, Headey D, Masters WA. Misreporting Month of Birth: Diagnosis and Implications for Research on Nutrition and Early Childhood in Developing Countries. *Demography* 2019;56(2):707-28. doi: 10.1007/s13524-018-0753-9
36. Malviya A, Ahirwar AK, Chandra Tripathi S, et al. COVID-19: a review on SARS-CoV-2 origin, epidemiology, virology, clinical manifestations and complications with special emphasis on adverse outcome in Bhopal Gas Tragedy survivor. *Horm Mol Biol Clin Investig* 2021;42(1):63-68. doi: 10.1515/hmbci-2020-0070 [published Online First: 2021/02/19]
37. Dore B. Covid-19: collateral damage of lockdown in India. *BMJ* 2020;369:m1711. doi: 10.1136/bmj.m1711 [published Online First: 2020/05/02]

For peer review only - http://bmjopen.bmj.com/site/about/guidelines.xhtml

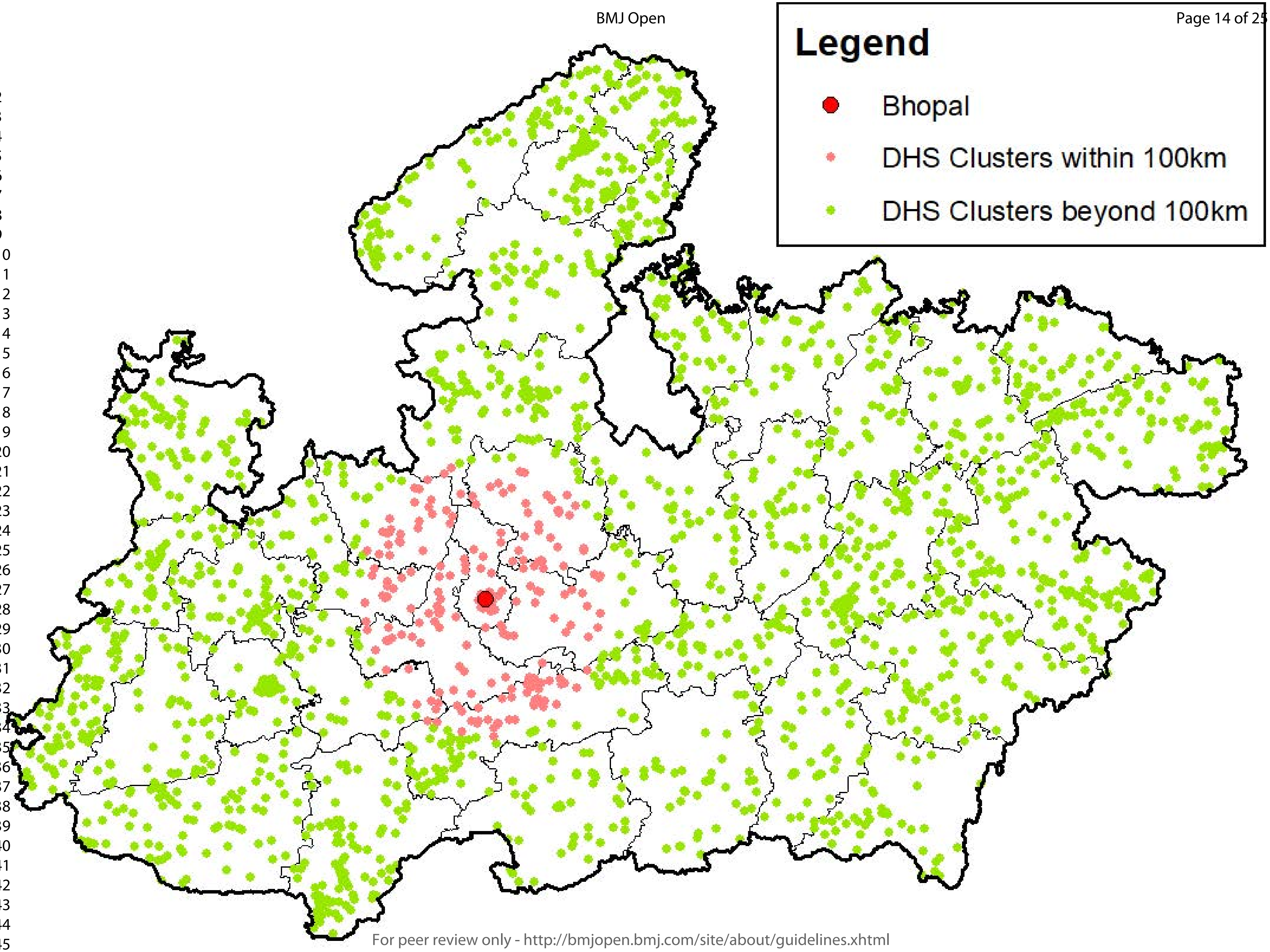

For peer review only - http://bmjopen.bmj.com/site/about/guidelines.xhtml

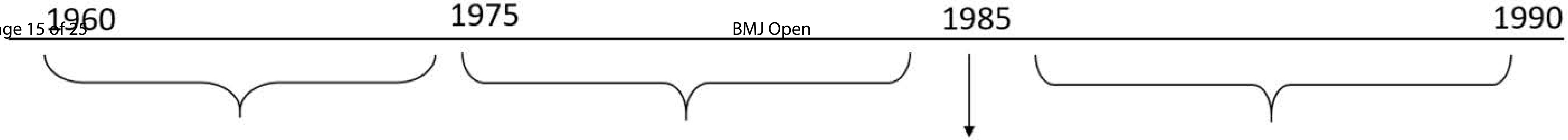

1960-1974	1975-1984	1985	1986-1990
Reference Cohort	T1	T2	T3
>10 years old at the time of Bhopal tragedy	0-10 years old at the time of Bhopal tragedy	Fetuses at the time of Bhopal tragedy	Not yet conceived at the time of Bhopal tragedy

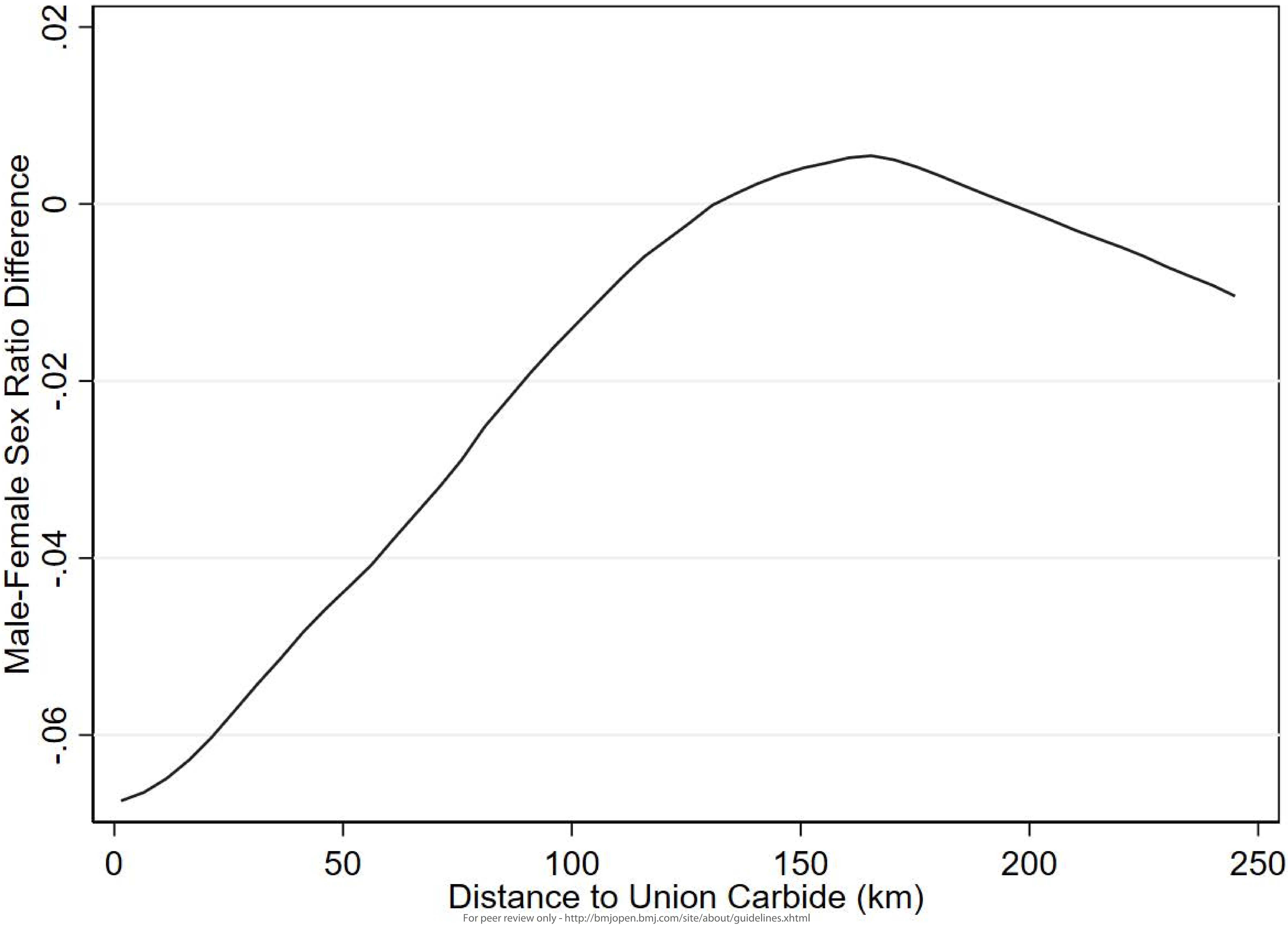
.02
0
-.02
-.04
-.06
Male-Female Sex Ratio Difference
0
50
100
150
200
250
Distance to Union Carbide (km)

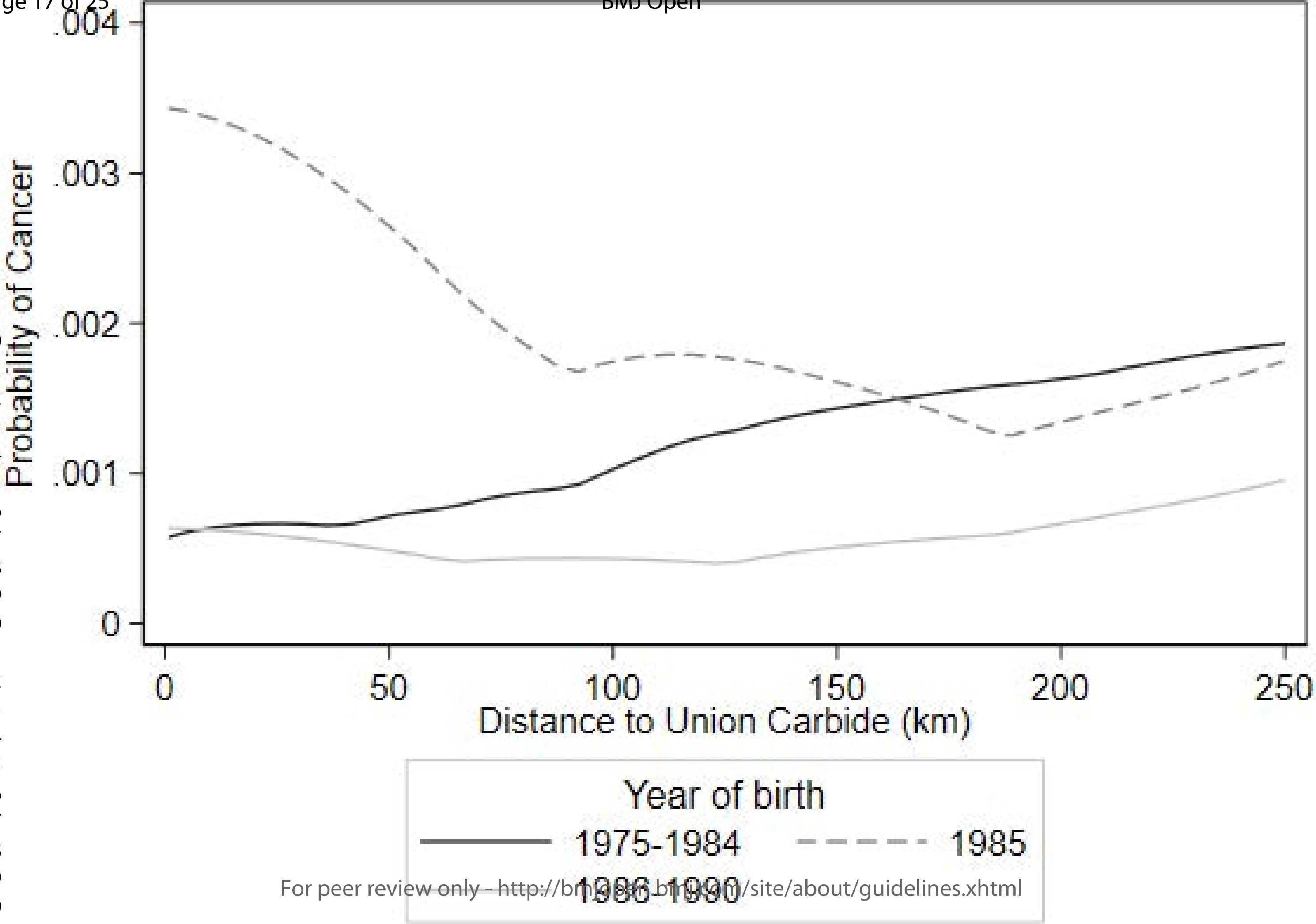

For peer review only - http://bmjopen.bmj.com/site/about/guidelines.xhtml

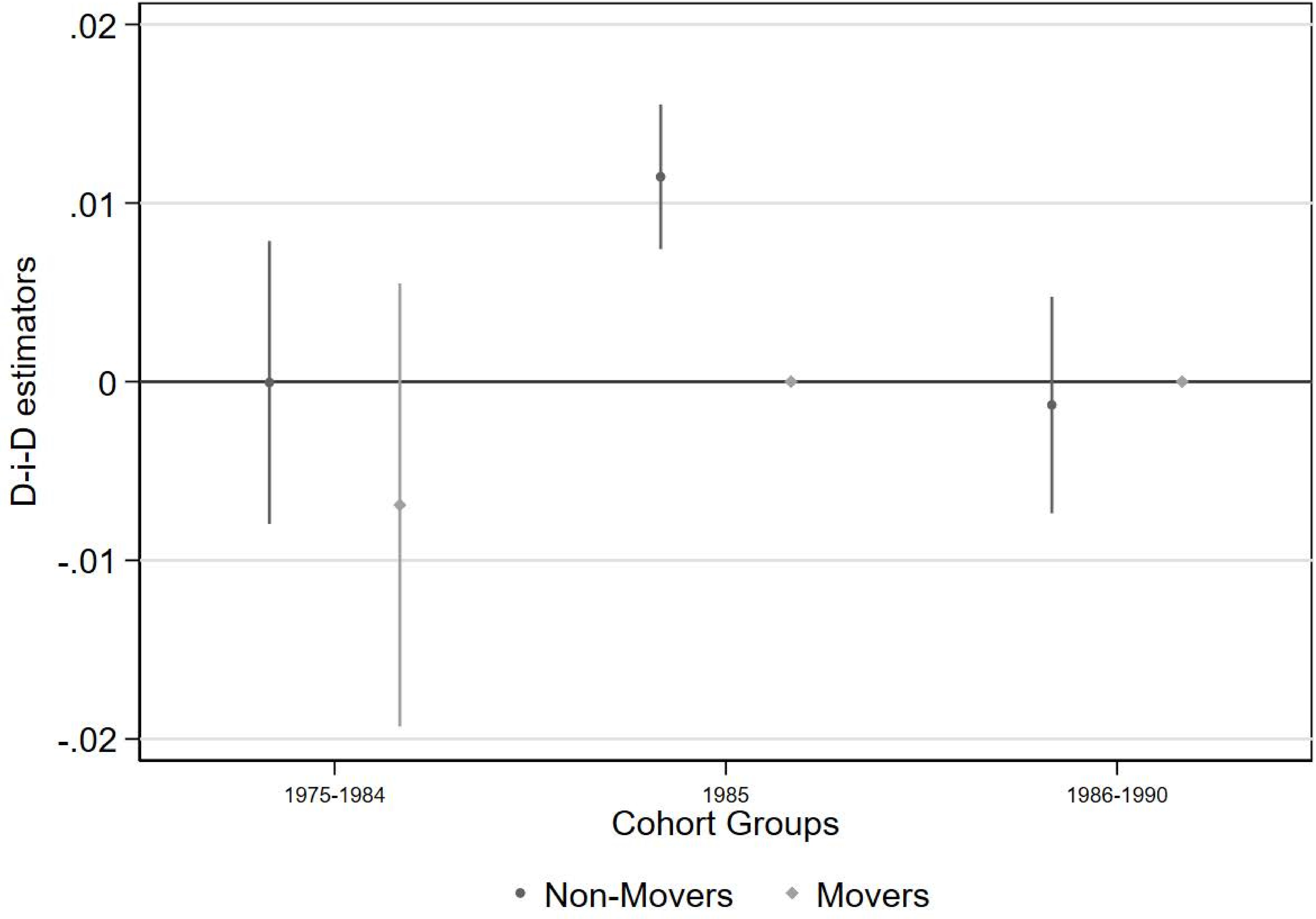
.02
.01
0
-.01
-.02
D-i-D estimators
1975-1984
1985
1986-1990
Cohort Groups
Non-Movers
Movers

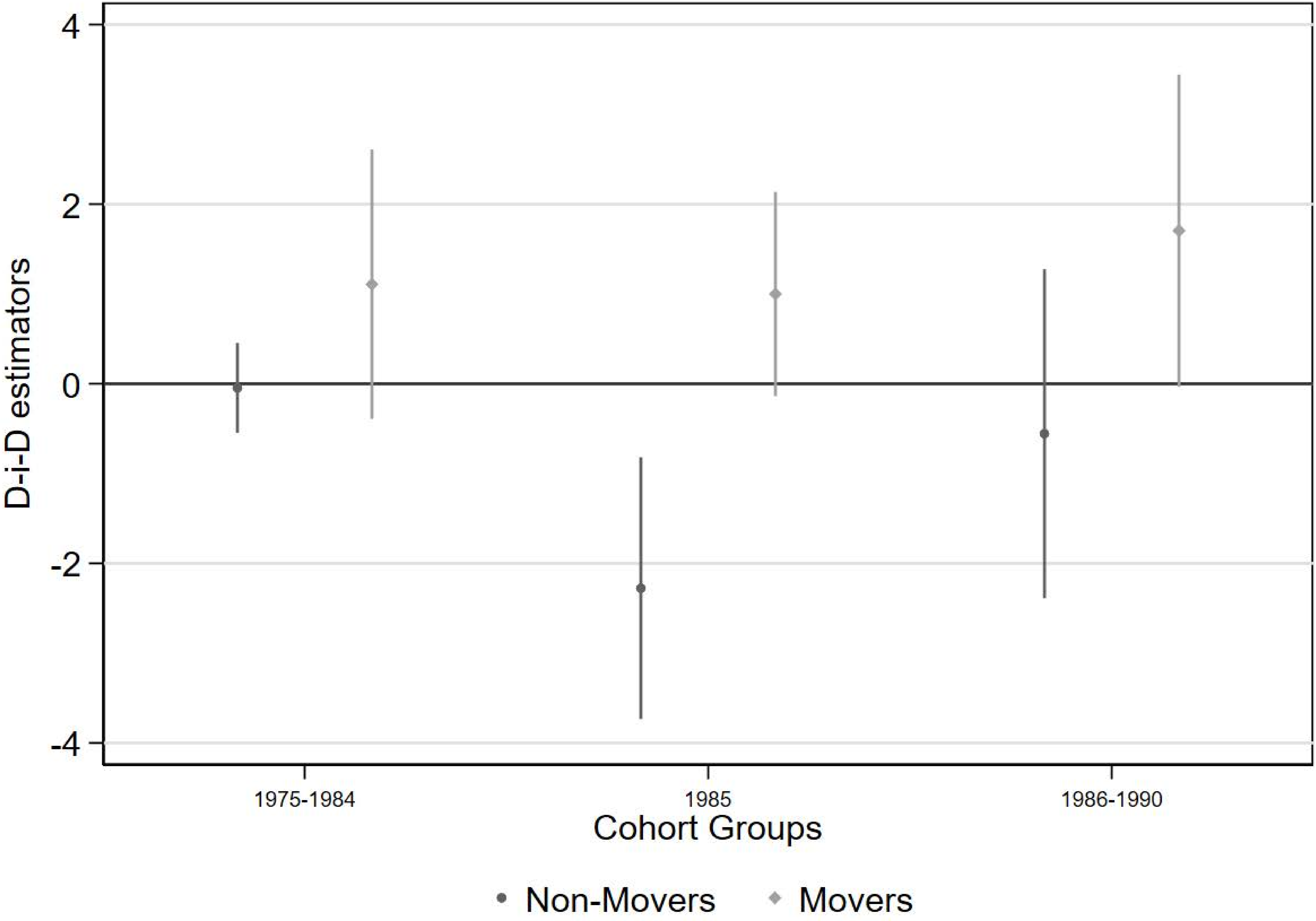
4
2
0
-2
-4
D-i-D estimators
1975-1984
1985
1986-1990
Cohort Groups
Non-Movers
Movers

In Utero Exposure to Industrial Disasters: A Case Study of the Bhopal Gas Tragedy

Supplemental Material

Gordon C. McCord[1]

Prashant Bharadwaj[2]

Lotus McDougal[3]

Arushi Kaushik[4]

Anita Raj[5]

Figure S1 uses the Indian Human Development Survey (IHDS) in a robustness check to test whether mothers of those who were in utero at the time of the BGD are different from those of other cohorts. The IHDS-I was conducted in 2004-05 and is representative at the district level. It is the only other survey apart from the DHS that collects health information for India. We compare mothers' education level and several (self-reported) health parameters, such as the probability of reporting to be suffering from a heart disease, high blood pressure, diabetes, asthma, tuberculosis, leprosy, cancer and mental illness (n=1,792).

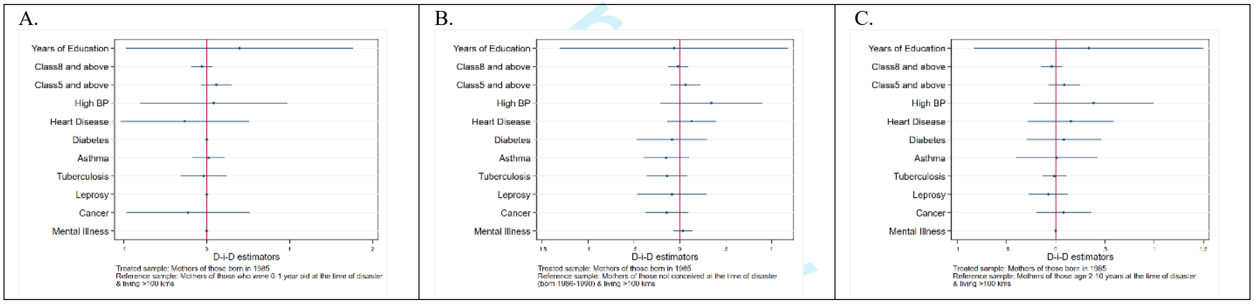

Figure S1: Characteristics of mothers of in utero cohort compared to others

The figure shows difference-in-difference estimates for various outcome variables for the mothers of those who were in utero (i.e, born in 1985). These estimates are obtained from regression equation similar to eq 1 where the control group includes mothers of non-mover men who were born between 1984 (i.e, <1 year old at the time of BGD; panel A), non-mover men who were born between 1975-1983 (i.e, 2-10 years old at the time of BGD; panel B) and non-mover men who were born between 1986-1990 (i.e, not conceived at the time of BGD; panel C) and are living > 100kms away from Union Carbide plant. The figures depict estimates with 95% CI.

[1] Corresponding author. gmccord@ucsd.edu, School of Global Policy and Strategy, UC San Diego
[2] Department of Economics, UC San Diego
[3] Center on Gender Equity and Health, Department of Medicine, UC San Diego
[4] Department of Economics, UC San Diego
[5] Center on Gender Equity and Health, Department of Medicine, UC San Diego

For peer review only - http://bmjopen.bmj.com/site/about/guidelines.xhtml

Table S1: Linear probability models (LPM) for employment disability among men living in Madhya Pradesh in 1999 within 100km of Bhopal, compared to men of the same cohort more than 100km from Bhopal.

	Prob(Reported Employment Disability)		
	(1)	(2)	(3)
	Pooled	Non-Movers	Movers
1986-1990	-0.00109	-0.00131	0
	(0.00257)	(0.00297)	(5.63e-11)
1985	0.0111***	0.0115***	0
	(0.00216)	(0.00198)	(9.65e-11)
1975-1984	-0.000632	-4.65e-05	-0.00690
	(0.00344)	(0.00388)	(0.00608)
1960-1974	Reference	Reference	Reference
Observations	13,369	12,129	1,240
R-squared	0.003	0.003	0.024
Cohort FEs	Yes	Yes	Yes
Control Mean	0.00377	0.00426	0

Robust standard errors in parentheses

*** $p<0.01$, ** $p<0.05$, * $p<0.1$

Notes: The cohort of men born in 1985 within 100 km of Bhopal has a higher likelihood of reporting employment disability compared to other cohorts and to those born in Madhya Pradesh beyond 100 km from Bhopal (1), with effect most pronounced among men who have never moved (2) instead of those who have moved (3). Estimates are from ordinary least squares regressions as in Equation (I) with controls consisting of fixed effects for year of birth cohort and whether the DHS cluster is within 100 km of Bhopal. Standard errors are two-way clustered at the district and year-of-birth levels.

For peer review only - http://bmjopen.bmj.com/site/about/guidelines.xhtml

Table S2: Linear probability models (LPM) for employment disability among men who have never moved living in Madhya Pradesh in 1999 at various distances from Bhopal, compared to men of the same cohort more than 100km from Bhopal.

	Prob(Employment Disability) among Non-Movers, by Treatment Radius		
	(1)	(2)	(3)
	100 km	75 km	50 km
1986-1990	-0.00131	-0.00183	-0.00045
	(0.00297)	(0.00390)	(0.00445)
1985	0.0115***	0.0138***	0.0203**
	(0.00198)	(0.00217)	(0.0078)
1975-1984	-4.65e-05	-0.00049	-0.00004
	(0.00388)	(0.00314)	(0.00294)
1960-1974	Reference	Reference	Reference
Observations	12,129	11,799	11,100
R-squared	0.003	0.002	0.003
Cohort FEs	Yes	Yes	Yes
Control Mean	0.00426	0.00437	0.0043

Robust standard errors in parentheses

*** p<0.01, ** p<0.05, * p<0.1

Notes: Estimates are from ordinary least squares regressions as in Equation (I) with controls consisting of fixed effects for year of birth cohort and whether the DHS cluster is within the specified distance from Bhopal. Standard errors are two-way clustered at the district and year-of-birth levels.

For peer review only - http://bmjopen.bmj.com/site/about/guidelines.xhtml

Table S3: Regression results on educational attainment among men born in Madhya Pradesh within 100km of Bhopal, compared to men of the same cohort more than 100km from Bhopal.

	Years of Education Completed		
	(1)	(2)	(3)
	Pooled	Non-Movers	Movers
1986-1990	0.221	-0.557	1.704*
	(0.601)	(0.897)	(0.851)
1985	-0.577	-2.276***	0.999*
	(0.376)	(0.714)	(0.557)
1975-1984	0.255	-0.0471	1.108
	(0.279)	(0.245)	(0.734)
1960-1974	Reference	Reference	Reference
Observations	7,045	5,014	2,031
R-squared	0.048	0.055	0.047
Cohort FEs	Yes	Yes	Yes
Control Mean	5.833	5.561	6.600

Robust standard errors in parentheses

*** $p<0.01$, ** $p<0.05$, * $p<0.1$

Notes: The cohort of men born in 1985 within 100 km of Bhopal has fewer years of educational attainment compared to other cohorts and to those born in Madhya Pradesh beyond 100 km from Bhopal (1), with effect most pronounced among men who have never moved (2) instead of those who have moved (3). Estimates are from ordinary least squares regressions as in Equation (I) with controls consisting of fixed effects for year of birth cohort and whether the DHS cluster is within 100 km of Bhopal. Standard errors are two-way clustered at the district and year-of-birth levels.

For peer review only

For peer review only - http://bmjopen.bmj.com/site/about/guidelines.xhtml

Table S4: Regression results on educational attainment among non-mover men born in Madhya Pradesh at varying distances from Bhopal, compared to other men of the same cohort beyond 100 km from Bhopal.

	Years of Education Completed among Non-Movers, by Treatment Radius		
	(1)	(2)	(3)
	100 km	75 km	50 km
1986-1990	-0.557	-0.356	-0.264
	(0.897)	(0.682)	(0.454)
1985	-2.276***	-2.253**	-0.316
	(0.714)	(0.983)	(2.200)
1975-1984	-0.0471	-0.371	-0.900
	(0.245)	(1.292)	(1.970)
1960-1974	Reference	Reference	Reference
Observations	5,014	4,830	4,702
R-squared	0.055	0.055	0.053
Cohort FEs	Yes	Yes	Yes
Control Mean	5.561	5.584	5.643

Robust standard errors in parentheses

*** p<0.01, ** p<0.05, * p<0.1

Notes: Estimates are from ordinary least squares regressions as in Equation (I) with controls consisting of fixed effects for year of birth cohort and whether the DHS cluster is within the specified distance from Bhopal. Standard errors are two-way clustered at the district and year-of-birth levels.

For peer review only

For peer review only - http://bmjopen.bmj.com/site/about/guidelines.xhtml

STROBE Statement—Checklist of items that should be included in reports of ***cohort studies***

	Item No	Recommendation	Page No
**Title and abstract**	1	(*a*) Indicate the study's design with a commonly used term in the title or the abstract	1
		(*b*) Provide in the abstract an informative and balanced summary of what was done and what was found	1
**Introduction**			
Background/rationale	2	Explain the scientific background and rationale for the investigation being reported	2
Objectives	3	State specific objectives, including any prespecified hypotheses	3
**Methods**			
Study design	4	Present key elements of study design early in the paper	4
Setting	5	Describe the setting, locations, and relevant dates, including periods of recruitment, exposure, follow-up, and data collection	2-3
Participants	6	(*a*) Give the eligibility criteria, and the sources and methods of selection of participants. Describe methods of follow-up<br>(*b*) For matched studies, give matching criteria and number of exposed and unexposed	3
Variables	7	Clearly define all outcomes, exposures, predictors, potential confounders, and effect modifiers. Give diagnostic criteria, if applicable	3-4
Data sources/ measurement	8*	For each variable of interest, give sources of data and details of methods of assessment (measurement). Describe comparability of assessment methods if there is more than one group	3
Bias	9	Describe any efforts to address potential sources of bias	10
Study size	10	Explain how the study size was arrived at	3
Quantitative variables	11	Explain how quantitative variables were handled in the analyses. If applicable, describe which groupings were chosen and why	3
Statistical methods	12	(*a*) Describe all statistical methods, including those used to control for confounding<br>(*b*) Describe any methods used to examine subgroups and interactions<br>(*c*) Explain how missing data were addressed<br>(*d*) If applicable, explain how loss to follow-up was addressed<br>(*e*) Describe any sensitivity analyses	5
**Results**			
Participants	13*	(a) Report numbers of individuals at each stage of study—eg numbers potentially eligible, examined for eligibility, confirmed eligible, included in the study, completing follow-up, and analysed<br>(b) Give reasons for non-participation at each stage<br>(c) Consider use of a flow diagram	3
Descriptive data	14*	(a) Give characteristics of study participants (eg demographic, clinical, social) and information on exposures and potential confounders<br>(b) Indicate number of participants with missing data for each variable of interest<br>(c) Summarise follow-up time (eg, average and total amount)	3
Outcome data	15*	Report numbers of outcome events or summary measures over time	4

For peer review only

Main results	16	(*a*) Give unadjusted estimates and, if applicable, confounder-adjusted estimates and their precision (eg, 95% confidence interval). Make clear which confounders were adjusted for and why they were included<br>(*b*) Report category boundaries when continuous variables were categorized<br>(*c*) If relevant, consider translating estimates of relative risk into absolute risk for a meaningful time period	7
Other analyses	17	Report other analyses done—eg analyses of subgroups and interactions, and sensitivity analyses	7-9
**Discussion**			
Key results	18	Summarise key results with reference to study objectives	9
Limitations	19	Discuss limitations of the study, taking into account sources of potential bias or imprecision. Discuss both direction and magnitude of any potential bias	10
Interpretation	20	Give a cautious overall interpretation of results considering objectives, limitations, multiplicity of analyses, results from similar studies, and other relevant evidence	10
Generalisability	21	Discuss the generalisability (external validity) of the study results	10
**Other information**			
Funding	22	Give the source of funding and the role of the funders for the present study and, if applicable, for the original study on which the present article is based	1

*Give information separately for exposed and unexposed groups.

Note: An Explanation and Elaboration article discusses each checklist item and gives methodological background and published examples of transparent reporting. The STROBE checklist is best used in conjunction with this article (freely available on the Web sites of PLoS Medicine at http://www.plosmedicine.org/, Annals of Internal Medicine at http://www.annals.org/, and Epidemiology at http://www.epidem.com/). Information on the STROBE Initiative is available at http://www.strobe-statement.org.

For peer review only

BMJ Open

Long-term Health and Human Capital Effects of In Utero Exposure to an Industrial Disaster: A Spatial Difference-in-Difference Analysis of the Bhopal Gas Tragedy

Journal:	*BMJ Open*
Manuscript ID	bmjopen-2022-066733.R2
Article Type:	Original research
Date Submitted by the Author:	04-Apr-2023

Complete List of Authors:	McCord, Gordon C ; University of California San Diego, School of Global Policy and Strategy<br>Bharadwaj, Prashant; University of California San Diego, Department of Economics<br>McDougal, Lotus; University of California San Diego, Department of Medicine<br>Kaushik, Arushi; University of California San Diego, Department of Economics<br>Raj, Anita; University of California San Diego, Department of Medicine

**Primary Subject Heading**:	Public health
Secondary Subject Heading:	Global health
Keywords:	PUBLIC HEALTH, EPIDEMIOLOGY, HEALTH ECONOMICS

SCHOLARONE™
Manuscripts

I, the Submitting Author has the right to grant and does grant on behalf of all authors of the Work (as defined in the below author licence), an exclusive licence and/or a non-exclusive licence for contributions from authors who are: i) UK Crown employees; ii) where BMJ has agreed a CC-BY licence shall apply, and/or iii) in accordance with the terms applicable for US Federal Government officers or employees acting as part of their official duties; on a worldwide, perpetual, irrevocable, royalty-free basis to BMJ Publishing Group Ltd ("BMJ") its licensees and where the relevant Journal is co-owned by BMJ to the co-owners of the Journal, to publish the Work in this journal and any other BMJ products and to exploit all rights, as set out in our licence.

The Submitting Author accepts and understands that any supply made under these terms is made by BMJ to the Submitting Author unless you are acting as an employee on behalf of your employer or a postgraduate student of an affiliated institution which is paying any applicable article publishing charge ("APC") for Open Access articles. Where the Submitting Author wishes to make the Work available on an Open Access basis (and intends to pay the relevant APC), the terms of reuse of such Open Access shall be governed by a Creative Commons licence – details of these licences and which Creative Commons licence will apply to this Work are set out in our licence referred to above.

Other than as permitted in any relevant BMJ Author's Self Archiving Policies, I confirm this Work has not been accepted for publication elsewhere, is not being considered for publication elsewhere and does not duplicate material already published. I confirm all authors consent to publication of this Work and authorise the granting of this licence.

For peer review only

Long-term Health and Human Capital Effects of *In Utero* Exposure to an Industrial Disaster: A Spatial Difference-in-Difference Analysis of the Bhopal Gas Tragedy

Gordon C. McCord[1]

Prashant Bharadwaj[2]

Lotus McDougal[3]

Arushi Kaushik[4]

Anita Raj[5]

[1] Corresponding author. gmccord@ucsd.edu, School of Global Policy and Strategy, UC San Diego
[2] Department of Economics, UC San Diego
[3] Center on Gender Equity and Health, Department of Medicine, UC San Diego
[4] Department of Economics, UC San Diego
[5] Center on Gender Equity and Health, Department of Medicine, UC San Diego

For peer review only

Abstract

Objectives: Globalization and industrialization can increase economic opportunity for low- and middle-income nations, but these processes may also increase industrial accidents and harm workers. This paper examines the long-term, cohort-based health effects of the Bhopal Gas Disaster (BGD), one of the most serious industrial accidents in history.
Design: This retrospective analysis uses geolocated data on health and education from India's National Family Health Survey-4 (NFHS-4) and the 1999 Indian Socio-Economic Survey [NSSO-1999] to examine the health effects of exposure to the BGD among men and women aged 15-49 living in Madhya Pradesh in 2015-16 (women n=40,786; men n=7,031 [NFHS-4] and n=13,369 [NSSO-1999]), as well as their children (n=1,260). A spatial difference-in-differences strategy estimated the relative effect of being in utero near Bhopal relative to other cohorts and to those further from Bhopal separately for each dataset.
Results: We document long-term, intergenerational impacts of the BGD, showing that men who were in utero at the time were more likely to have a disability that affected their employment 15 years later, and had higher rates of cancer and lower educational attainment over 30 years later. Changes in the sex ratio among children born in 1985 suggest an effect of the BGD up to 100km from the accident.
Conclusions: These results indicate social costs stemming from the BGD that extend far beyond the mortality and morbidity experienced in the immediate aftermath. Quantifying these multigenerational impacts are important for policy consideration. Moreover, our results suggest that the BGD affected people across a substantially more widespread area than has previously been demonstrated.

Ethical approval: All data used in this study are publicly available and de-identified. Ethical approval for NFHS-4 data collection was provided by the International Institute for Population Sciences and ICF. Ethical approval for the 1999 NSSO Socio-Economic Survey was provided by the Indian National Sample Survey Office. Ethical exemption for this analysis was provided by the University of California San Diego IRB.

For peer review only - http://bmjopen.bmj.com/site/about/guidelines.xhtml

Transparency statement: The authors affirm that the manuscript is an honest, accurate, and transparent account of the study being reported; that no important aspects of the study have been omitted.

Funding: AR and LM received funding from the Bill and Melinda Gates Foundation (INV-008648).

Strengths and Limitations of this Study

- This study uses different birth cohorts and a geographic difference-in-difference strategy to attribute causality of subsequent health and social consequences to the Bhopal Gas Disaster.
- The design nets out any difference in outcomes that arise due to time invariant aspects of being close to Bhopal, as well as aspects of belonging to a specific birth cohort.
- As with any ecological exposure assignment, the cohort of in utero children we assign to exposure will include a range of actual exposure to MIC gas.
- Mortality can affect the composition of the population observed post-facto; if the weakest children in utero were more likely to die from the incident, then our estimates could be considered a lower bound.
- The long-term consequences that we estimate could be the result of both direct effects from exposure as well as lack of subsequent mitigation of the effects through health, disability and education services.

Introduction

Globalization and industrialization can increase economic opportunity for low- and middle-income nations, but recent cross-national evidence indicates that these processes may also be increasing industrial accidents and harming the workers of these nations.[1] Evidence from India suggests high numbers of industrial accidents, with the highest associated attributable deaths in the world.[2 3] Despite recommendations for improved protections for workers and industry in India,[3] the Ministry of Environment, Forest and Climate Change has proposed the Environmental Impact Assessment (EIA) notification 2020, which will roll back environmental and worker protections in the country. Critics, including United Nations experts, have stated that this rollback violates the safety and health of workers and the environment and with potential impact on subsequent generations.[4-6] Unfortunately, little research is available on these multigenerational impacts, and inadequate recognition of these far-reaching repercussions can diminish the memory of such tragedies for policy consideration. This research provides insight into these impacts on children born to women survivors of one of the worst industrial disasters in history, the Bhopal Gas Disaster.

The Bhopal Gas Disaster (BGD) occurred in December 1984 and involved a methyl isocyanate (MIC) gas leak at a Union Carbide pesticide plant near the city of Bhopal, in Madhya Pradesh, India. The leak spread toxic gas in approximately a 7 km radius around the plant, exposing more than half a million people and resulting in up to 30,000 deaths in the region.[7] There is a broad spectrum of serious long-term and chronic health consequences for hundreds of thousands of survivors, including children, manifesting across multiple systems (e.g. respiratory, neurologic, musculoskeletal, ophthalmic, endocrine).[7-10] These impacts may be the tip of the iceberg however, given that MIC toxins affected groundwater[11] and the reproductive health and other health outcomes of exposed women,[8] factors suggesting that generations not exposed to the toxic gas directly may nevertheless suffer adverse health and social impacts of the BGD event. Potential in utero effects cannot be understated. Studies document a 4-fold increase in the rate of

For peer review only - http://bmjopen.bmj.com/site/about/guidelines.xhtml

miscarriage following the gas leak, as well as increased risk for stillbirth and neonatal mortality.[9 10] Decades after the disaster, menstrual abnormalities and premature menopause have emerged as common problems among exposed women and their female offspring.[9] MIC has also been shown to damage human chromosomes.[12] Early clinical studies on the gas-affected population showed signs of increased chromosomal aberrations that could manifest as cancer,[13] though population-level changes in cancer rates 8 years after the accident were suggestive but not significant.[14]

This paper estimates the long-term health (specifically, adult cancer rates and disability) and human capital impacts (educational attainment) on individuals who were exposed to the BGD in utero or as children in 1984. Understanding the long-term impacts of industrial disasters on children, especially those in utero at the time of the disaster, is important for several key reasons. First, while the immediate damage to children in the form of direct health impacts is perhaps easier to assess, the long-term, health-related damages could take years to manifest and might not be part of the legal damages considered. Second, economic research over the last few decades has shown that interventions that improve outcomes for children have high rates of return;[15] to this second point, we include education as an important indicator of socioeconomic impact on these children, particularly as these secondary effects may compound health impacts. Our findings offer timely information to inform debate on potential rollbacks of policies related to social and environmental protections attached to industrialization in India and elsewhere.

Methods

Data

The principal data source is the fourth round of the Demographic and Health Surveys (DHS) in India, which was conducted between 2015-2016 (also called National Family Health Survey, or NFHS-4, in India).[16] The DHS/NFHS-4 is a high-quality household survey that provides detailed information on health at the individual level, and is representative at both the national and state levels. All interviews in Madhya Pradesh were completed in 2015. For the first analysis focusing on differences in the sex ratio, the analytic sample includes children born between 1981-1985 to women who were interviewed in Madhya Pradesh and who lived within 250 km of Bhopal (n=1,260 births). The analytic sample for the analysis of cancer incidence includes men and women who were interviewed in Madhya Pradesh and were born between 1960 and 1990 (n=7,031 men and 40,786 women).

A second dataset that we use is the Integrated Public Use Microdata Series (IPUMS) from India for the year 1999, which presents harmonized data from the 1999 Socio-Economic Survey conducted by the National Sample Survey Organization (NSSO).[17] These data contain employment information for a representative sample, but only identifies the district of the respondent. This is the most recent NSSO survey in which respondents were asked how long they had been living at their current residence, a crucial question for this analysis. The survey sample includes people from ages 6-64, thus including those who were in utero at the time of the BGD. The analytic sample for this analysis includes men interviewed in Madhya Pradesh and born between 1960 and 1990 (n=13,369)

The unit of observation in all analyses is the individual. The DHS/NFHS-4 and NSSO datasets could not be merged at the individual level, as the same respondents were not interviewed in each survey. In order to preserve the more precise geolocation of the NHFS-4, the two datasets were analyzed separately.

For peer review only - http://bmjopen.bmj.com/site/about/guidelines.xhtml

Patient and Public Involvement
Since DHS data are anonymized, direct involvement with study subjects is not possible. Once published, results will be disseminated to the public in Bhopal through a letter to local newspaper media.

Measures
For the analysis of children born to women surveyed by the NFHS-4, the outcome of interest was the ratio of male-to-female births.

For men, the outcomes of interest were cancer rates and educational attainment (from the NFHS-4) and employment disability (from the NSSO). Cancer was assessed as a general question "Do you currently have cancer", and educational attainment was assessed as the number of years that an individual attended school. Of the sample of men, 17 out of 7,031 with nonmissing response reported to have cancer, and the average number of years of education was 7.0 with a standard deviation of 5.1. We use the question on whether "the respondent was economically inactive because of disabilities or...other health-related reasons" to classify the person as suffering from employment disability. Forty-six men in the sample of 13,369 report to be suffering from employment disability. Given the prevalence of child labor, note that the employment-related questions of the survey are asked of all respondents, including children. Analyses assessing *movers* vs. *non-movers* are necessarily restricted to men, since patrilocality in India means that most women move to their husband's house after marriage.[18] As 78% of Indian women aged 15-49 have married,[16] the sample size of non-mover females is both small and non-representative, and thus cannot be considered in this analysis.

For all analyses, distance from the Union Carbide plant was the primary predictor. The NFHS-4 collected the latitude and longitude of each respondent's sampling cluster (henceforth, DHS clusters). The DHS clusters are villages or hamlets in rural areas and blocks or census tracts in urban areas; thus, they are much smaller geographical units than administrative units such as districts. These coordinates enable the calculation of distance from the Union Carbide plant in Bhopal for each respondent, within a privacy displacement radius of 2 km (urban) or 5-10 km (rural) (Figure 1).

Figure 1: Madhya Pradesh and DHS cluster locations around Bhopal

In the case of the district-level NSSO data, we measure distance from the Union Carbide plant to the nearest border of the district. For both datasets, we use the distance from the plant to form the treatment and control groups, since exposure to the toxic MIC gas would be more muted at greater distances.

Analysis

The analysis uses a spatial difference-in-differences strategy, where distance from the Union Carbide plant (we also refer to this as "distance from Bhopal") lends the spatial dimension. We compare people living close to Bhopal to those far away from Bhopal, and people who were in utero at the time of the BGD to those who were older or not yet conceived at the time of BGD. Under some standard assumptions,[19] these estimates net out any difference in outcomes that arise due to *time invariant* aspects of being close to Bhopal, as well as aspects of belonging to a specific birth cohort. The estimation also nets out the effect of other programs that might have been occurring during these years across multiple birth cohorts, such as the Universal Immunization Program.

All analyses were conducted using birth year cohorts (Figure 2). The reference cohort includes those born between 1960-1974 (i.e. respondents who were more than 10 years old at the time of the BGD). Three other cohorts are considered: young children below 10 years old at the time of the BGD (i.e., respondents

For peer review only - http://bmjopen.bmj.com/site/about/guidelines.xhtml

born between 1975-1984), fetuses (i.e. respondents born in 1985) and children not yet conceived at the time of BGD (i.e. respondents born between 1986-1990). This last cohort can be considered a placebo test, as we expect effects of the BGD on children not yet conceived to be null or significantly smaller than for cohorts that were alive or in utero.

Figure 2: Description of all cohorts used in empirical estimations.

It is worth noting that all four cohorts have been affected by the disaster, including our reference cohort. Therefore, while older cohorts are being used as a reference category, our results speak to the *relative* impacts on those who were in utero at the time of the disaster.

Linear regression models were used to estimate the relationship between proximity to Bhopal, birth cohort, and outcomes of interest, with the basic specification as follows:

$$\text{(I)} \qquad Y_{itc} = \alpha_1 Bhopal_c + \beta_1 * T1_t * Bhopal_c + \beta_2 * T2_t * Bhopal_c + \beta_3 * T3_t * Bhopal_c + \text{Cohort FE} + \varepsilon_{itc}$$

where Y_{itc} is the outcome of interest for person *i* born in year *t* and living in location *c*. We consider three health and education outcomes, namely: the probability of suffering from cancer, the probability of suffering from employment disability, and years of education completed. $Bhopal_c$ takes the value of 1 if the respondent *i* is living near Bhopal (we define "near" Bhopal as being within 100kms following spatial patterns in sex ratios explained in Figure 3) and 0 otherwise. $T1_t$ is a dummy variable equal to 1 if the respondent was born between 1975-1984 (cohort of ages 0-10 at the time of BGD) and 0 otherwise. $T2_t$ is a dummy variable equal to 1 if the respondent was born in 1985 (in utero cohort) and 0 otherwise. $T3_t$ is a dummy variable equal to 1 if the respondent was born between 1986-1990 (cohort not yet conceived at the time of the BGD) and 0 otherwise. The omitted category consists of respondents who were older than 10 years at the time of the disaster and are living more than 100 kms away from Bhopal. Cohort FE refers to birth year fixed effects to standardize the outcome variable across ages. Standard errors are two-way clustered by district and birth year in order to account for cross-sectional and serial autocorrelation within district, as well as covariate shocks across all people in the same birth cohort (such as changes in food prices).

β_1, $\beta_{2,}$ and β_3 respectively capture the effect of the BGD on young children below 10 years old, fetuses and not-yet-conceived children relative to those who were older than 10 at the time of the BGD. The spatial difference-in-difference compares children in a cohort living near Bhopal to children in the same cohort living far from Bhopal. In order to interpret β_1, $\beta_{2,}$ and β_3 as the causal effect of the BGP, the underlying identifying assumption is that in the absence of the disaster the cohorts near and far from Bhopal would have evolved similarly. Note that α_1 captures the effect of living near Bhopal vis-a-vis far from Bhopal for the reference cohort (1965-1974). This difference captures time invariant differences in the two geographical locations.

Results

Sex ratio changes following the Bhopal Gas Disaster

The sex ratio of children born between 1981-1984 is markedly different from that of children born in 1985 (Figure 3). Women who lived within 100 km of Bhopal experienced a relative decrease in the birth of males in the 1985 cohort (64% of children born from 1981-1984 were male, a proportion that drops to 60% in 1985). This is consistent with male fetuses more affected by external stress.[20-22] Women living beyond 100km had no difference in the sex ratio across the 1981-1984 and the 1985 cohorts.

For peer review only - http://bmjopen.bmj.com/site/about/guidelines.xhtml

Figure 3. Difference in male-female sex ratio between 1985 and 1981-1984 cohorts among children born to women ages 15-49 in Madhya Pradesh, by distance to Union Carbide plant.

Adult Cancer Rates among Exposed Children

Similar to patterns seen with changes in the sex ratio, children who were born in 1985 (the year following the BGD) and who lived close to Bhopal during the disaster experience much higher rates of cancer as adults compared to adults who were born before or after the disaster and who lived further away from Bhopal (Figure 4). As in the case of differences in sex ratios, the abnormally high cancer rates appear to extend up to 100 km from Bhopal. Given the two spatial patterns in Figures 3 & 4, we use 100 km as the BGD exposure cutoff in our analysis.

Figure 4. Self-reported cancer incidence among men and women living in Madhya Pradesh in 2015, by distance to Union Carbide plant.

Among all men in the NFHS-4 analytic sample, estimates suggest that the 1975-1984 and 1986-1990 birth cohorts have statistically indistinguishable cancer rates compared to the 1960-1974 cohort, while men born within 100 km of Bhopal in 1985 have a 2.1 percentage point higher risk than the other cohorts (Table 1). This is an 8-fold higher cancer risk compared to the other cohorts.

Table 1: Probability of suffering from cancer among men born in Madhya Pradesh.

	Prob(Reports having Cancer)		
	(1)	(2)	(3)
	Pooled	Non-Movers	Movers
1986-1990	-0.00373	0.00170	-0.0141
	(0.00340)	(0.00147)	(0.0121)
1985	0.0209***	0.0624***	-0.00906
	(0.00591)	(0.00505)	(0.0113)
1975-1984	-0.00459	-0.00157	-0.00906
	(0.00355)	(0.00181)	(0.0113)
1960-1974	Reference	Reference	Reference
Observations	7,031	5,002	2,029
R-squared	0.007	0.012	0.014
Cohort FEs	Yes	Yes	Yes
Control Mean	0.00252	0.00228	0.00321
Robust standard errors in parentheses			
*** p<0.01, ** p<0.05, * p<0.1			

Notes: The cohort of men born in 1985 within 100 km of Bhopal has a higher likelihood of cancer compared to other cohorts and to those born in Madhya Pradesh beyond 100 km from Bhopal (1), with effect most pronounced among men who have never moved (2) instead of those who have moved (3). Estimates are from ordinary least squares regressions as in Equation (I) with controls consisting of fixed effects for year of birth cohort and whether the DHS cluster is within 100 km of Bhopal. Standard errors are two-way clustered at the district and year-of-birth levels.

For peer review only - http://bmjopen.bmj.com/site/about/guidelines.xhtml

Among men who have never moved, and were therefore residing within 100km of the Union Carbide plant during the BGD, the effect of being born in 1985 and near Bhopal is even stronger. These men have a 6.2 percentage point higher cancer risk in 2015 compared to the other cohorts and to those living more than 100 km from Bhopal.[1] This represents a 27-fold higher risk of cancer among adults who were in utero during the BGD, suggesting long-term health consequence of exposure, even while in the womb. Men who moved at some point before the survey in 2015/16 (and who were therefore less likely to be exposed to the BGD), and had no difference in cancer rates across cohorts.

Effects on Employment Disability

Men who were in utero during the BGD and who lived in districts with a border within 100 km of Bhopal are 1 percentage point more likely to report employment disability when they are surveyed as adults, compared to the older cohorts and those living further from Bhopal (Figure 5; Table S1). The effect increases to 2 percentage points among those living within 50 km of Bhopal (Table S2), consistent with higher intensity of exposure to MIC among those living closer to Bhopal.

Figure 5: Employment disability, compared to reference group of men born from 1960-1974 and living > 100 km from Bhopal. Bars represent 95% confidence intervals. Note that there were no individuals reporting employment disability in the 1985 and 1986-1990 cohorts among the *movers* subgroup.

Effects on Educational Attainment

Relative to other cohorts, men who were in utero during the BGD and who lived in districts with a border within 100 km of Bhopal attained approximately two fewer years of education, with the effects only evident among men who have never moved (Figure 6; Table S3). Limiting the treatment radius to 75 km yields the same estimated effect, while smaller distances yield no discernible effect (Table S4).

Figure 6: Years of education completed, compared to reference group of men born from 1960-1974 and living > 100 km from Bhopal.

Discussion

The Bhopal Gas Disaster was one the most serious industrial accidents in history, causing thousands of deaths at the time of the leak, and tens of thousands more deaths and serious health sequelae in the three decades since.[23 24] This analysis documents long-term, intergenerational impacts of the BGD, showing that men who were in utero at the time of the BGD were more likely to have a disability that affected their employment 15 years later, and had higher rates of cancer and lower educational attainment over 30 years later. Importantly, these results indicate social costs stemming from the BGD that extend far beyond the mortality and morbidity experienced in the immediate aftermath. Moreover, our results suggest that the BGD affected people across a substantially more widespread area than has previously been demonstrated:

[1] Given the presence of both fixed effects and interaction terms in the difference-in-differences framework, the linear probability model estimated with ordinary least squares is more appropriate than logistic regression. For completeness, we report the odds ratio for the 1985 cohort from a conditional logic model: 3.36 (0.1-111.8). While the estimation problems with logistic in the difference-in-differences context means we cannot interpret the magnitude, the sign is consistent with the results we report from ordinary least squares.

For peer review only - http://bmjopen.bmj.com/site/about/guidelines.xhtml

up to around 100 km (approximately 60 miles) from the site, as opposed to the 4.5 km radius that was considered exposed by public health officials and researchers after the disaster.[9 10 14 25] This study is also the first, to our knowledge, to use different birth cohorts to attribute causality of subsequent health and social consequences to the BGD.

We find that men currently living within 100 km of Bhopal and born in 1985 have an 8-fold higher risk of cancer than men of other birth cohorts; of those men, those who have never changed residence since the BGD have a 27-fold higher risk of cancer. While elevated cancer levels have been previously associated with MIC exposure,[14 26-28] our results identify long-term, birth-year based differences in cancer prevalence. This underscores the plausibility of multiple MIC exposure pathways, including teratogenic in utero exposure, and ongoing environmental exposure. Bhopal has ongoing cancer surveillance programs,[29] and it remains clear that those exposed to MIC require ongoing and careful monitoring, support and treatment.

Employment disability was one percentage point more likely among men who were in utero in districts within 100 km of Bhopal during the BGD than those born prior or more distal to the BGD. This is a meaningful impact since baseline employment disability rates are quite low (0.4%), and men's employment at the time of study was nearly universal at 98%.[16] In a context of high poverty, with nearly 40% of Madhya Pradesh's population living beneath the poverty line,[30] more than 90% informal employment nationally (thus lacking social protections and safety nets),[31] and household reliance on male income due to low levels of female labor force participation and a highly skewed gender wage gap,[16 32] this increase in disability or health-related economic inactivity risks substantial impact on the health, safety and well-being of affected families.

The finding that men who were in utero and within 100km of Bhopal during the BGD received more than two fewer years of education than other cohorts has tremendous human capital implications. This is a large impact both since the average number of years of education in the control group is only 5.6 years, and because education has such a direct association with subsequent wages and consumption.[33] We cannot ascertain whether the lower educational attainment is due to health or cognitive consequences of being exposed to the BGD in utero. Given that the BGD effect is much more evident in the 1985 cohort compared to older or younger children, it is unlikely that the effect operated through broader socioeconomic impacts or impacts on other family members that limited children's access to school. Regardless of the mechanism, the reduced educational attainment is evidence of long-term consequences of exposure to the accident that imply personal and social costs far beyond the immediate health impacts.

A few study limitations are worth outlining. First, as with any ecological exposure assignment, the cohort of in utero children we assign to exposure will include a range of actual exposure to MIC gas. The interpretation of our estimates would be the effect of average exposure in the population we define as treated. Secondly, our estimates are affected by mortality and migration. An important consequence of a disaster, whether natural or man-made, is that mortality can severely affect the composition of the population observed post-facto. In our empirical context, any mortality effect of the disaster that symmetrically affects all cohorts within Bhopal (without affecting people away from Bhopal) will not affect our estimates. As such, it is differential mortality across cohorts due to the BGD that would have compositional effects on our study sample. For example, if the weakest children in utero were more likely to die from the incident, then our estimates could be considered a lower bound.

Likewise, a frequent human response to a disaster is migration to safer areas. Again, it would only be differential migration in response to the BGD (different migration across cohorts and across people living close or far from Bhopal) that would create a problematic compositional difference between our treated cohort and the others. In fact, migration in this area was relatively low and researchers found that 91% of the population remained in the same area subsequent to 1984.[10] Our own estimates in Figure S1 suggest

For peer review only - http://bmjopen.bmj.com/site/about/guidelines.xhtml

that mothers of the in utero cohort are not significantly different from mothers of other consecutive cohorts on various educational and health dimensions.

It is also important to note that the long-term consequences that we estimate could be the result of both direct effects from exposure as well as lack of subsequent mitigation of the effects through health, disability and education services. While disentangling these forces is important from a policy perspective, in this paper we simply highlight the total combined effect of being exposed to such disasters as a child. As such, causal inference methods mitigate but do not eliminate the need to consider different routes of causation.

Work in other empirical contexts of industrial disasters should strive to estimate how effects on in utero children vary by trimester of pregnancy. Other kinds of shocks have been shown to vary in their effect by trimester,[34] however given data quality issues on long recall of month-of-birth in the DHS,[35] we do not attempt to estimate effects by trimester in this study.

Finally, cancer reporting in the data comes from self-reports, which could be subject to biases; there are also very few cases of self-reported cancer in the data. We think of our results on cancer as being consistent with the broader public health narrative on the consequences of the disaster, but more data through systematic cancer screening and place of birth information is key to firmly establish this link.

Understanding the short- and long-term damages caused by industrial disasters is key to gaining insight into the trade-offs involved in making regulatory decisions. It is also crucial from a policy response perspective if policy-makers wish to dedicate resources to mitigate harm done by such events or to compute legal damages. These concerns are particularly germane now, when there is evidence that the multiple health conditions suffered by many BGD survivors may make them more susceptible to the COVID-19 pandemic.[36 37] The evidence presented in this paper starkly highlights the long-term, intergenerational health and human capital effects of the BGD, and underscores the need for ongoing survivor support, as well as robust regulatory protections.

Funding Statement

AR and LM received funding from the Bill and Melinda Gates Foundation (INV-008648).

Declaration of interests

The authors declare no conflict of interest.

Author contributions

PB conceptualized the project, GCM and AK organized data and conducted analysis, all authors including LM and AR contributed equally to data interpretation, writing, review and editing. GCM and AK have verified the underlying data.

For peer review only - http://bmjopen.bmj.com/site/about/guidelines.xhtml

Ethics Statement

All data used in this study is publicly available and de-identified. Ethical approval for NFHS-4 data collection was provided by the International Institute for Population Sciences and ICF. Ethical approval for the 1999 NSSO Socio-Economic Survey was provided by the Indian National Sample Survey Office. Ethical exemption for this analysis was provided by the University of California San Diego IRB.

Data Sharing Statement

Replication code will be made available upon publication. The raw DHS survey data are subject to a user agreement and are available at available from the Demographics and Health Surveys Program at https://dhsprogram.com. The IPUMS data with the 1999 National Sample Survey are available from https://international.ipums.org/international/index.shtml.

The Corresponding Author has the right to grant on behalf of all authors and does grant on behalf of all authors, an exclusive licence (or non exclusive for government employees) on a worldwide basis to the BMJ Publishing Group Ltd to permit this article (if accepted) to be published in BMJ editions and any other BMJPGL products and sublicences such use and exploit all subsidiary rights, as set out in our licence.

For peer review only

For peer review only - http://bmjopen.bmj.com/site/about/guidelines.xhtml

References

1. Blanton RG, Peksen D. Dying for globalization? The impact of economic globalization on industrial accidents. *Social Science Quarterly* 2017;98(5):1487-502.
2. Nalapat S. Workplace disasters during lockdown put spotlight on India's patchy safety record -- explained with graphs. *Times Now News* 2020 September 7, 2020.
3. Sengupta A, Bandyopadhyay D, Van Westen C, et al. An evaluation of risk assessment framework for industrial accidents in India. *Journal of loss prevention in the process industries* 2016;41:295-302.
4. Explained: What is EIA 2020? How does it water down the existing policy? *The Week* 2020 August 10, 2020.
5. Explained Ideas: What ails with the draft EIA notification 2020? *The Indian Express* 2020 August 12, 2020.
6. UN experts to Govt of India: Environmental exemptions to industry unwarranted. 2020 September 4, 2020.
7. Sharma DC. Bhopal: 20 years on. *Lancet* 2005;365(9454):111-2. doi: 10.1016/S0140-6736(05)17722-8 [published Online First: 2005/01/12]
8. Ganguly BB, Mandal S, Kadam NN. Spectrum of health condition in methyl isocyanate (MIC)-exposed survivors measured after 30 years of disaster. *Environ Sci Pollut Res Int* 2018;25(5):4963-73. doi: 10.1007/s11356-017-0865-6 [published Online First: 2017/12/06]
9. Mishra PK, Samarth RM, Pathak N, et al. Bhopal Gas Tragedy: review of clinical and experimental findings after 25 years. *Int J Occup Med Environ Health* 2009;22(3):193-202. doi: 10.2478/v10001-009-0028-1 [published Online First: 2009/10/13]
10. Dhara VR, Dhara R. The Union Carbide disaster in Bhopal: a review of health effects. *Archives of Environmental Health: An International Journal* 2002;57(5):391-404.
11. Mishra B, Banerjee N. MIC leak disaster and environmental contamination: Time to act now. *Natl J Community Med* 2014;5:13-20.
12. Ennever FK, Rosenkranz HS. Evaluating the potential for genotoxic carcinogenicity of methyl isocyanate. *Toxicol Appl Pharmacol* 1987;91(3):502-05.
13. Malla T, Senthilkumar C, Sharma N, et al. Chromosome instability among Bhopal gas tragedy survivors. *Am Eur J Toxicol Sci* 2011;3:245-9.
14. Dikshit RP, Kanhere S. Cancer patterns of lung, oropharynx and oral cavity cancer in relation to gas exposure at Bhopal. *Cancer Causes Control* 1999;10(6):627-36.
15. Magnuson K, Duncan GJ. Can early childhood interventions decrease inequality of economic opportunity? *RSF: The Russell Sage Foundation Journal of the Social Sciences* 2016;2(2):123-41.

[dataset] 16. International Institute for Population Sciences (IIPS), ICF. National Family Health Survey (NFHS-4), 2015-16: India. Mumbai: IIPS, 2017.

[dataset] 17. National Sample Survey Office - M/o Statistics and Implementation (MOSPI), Government of India. India - Informal Non-Agricultural Enterprises Survey, July 1999 - June 2000, NSS 55th Round. In: NSSO, ed. Delhi, India, 1999-2000.

18. Khalil U, Mookerjee S. Patrilocal Residence and Women’s Social Status: Evidence from South Asia. *Economic Development and Cultural Change* 2019;67(2):401-38. doi: 10.1086/697584
19. Wooldridge J. Introductory Econometrics. 7th ed: Cengage Learning 2020.
20. Kraemer S. The fragile male. *BMJ (Clinical research ed)* 2000;321(7276):1609-12. doi: 10.1136/bmj.321.7276.1609
21. Hansen D, Moller H, Olsen J. Severe periconceptional life events and the sex ratio in offspring: follow up study based on five national registers. *BMJ (Clinical research ed)* 1999;319(7209):548-49. doi: 10.1136/bmj.319.7209.548
22. Mizuno R. The male/female ratio of fetal deaths and births in Japan. *Lancet* 2000;356(9231):738-9. doi: 10.1016/s0140-6736(00)02637-4

For peer review only - http://bmjopen.bmj.com/site/about/guidelines.xhtml

23. Broughton E. The Bhopal disaster and its aftermath: a review. *Environmental Health* 2005;4(1):6. doi: 10.1186/1476-069X-4-6
24. Odysseos L. The question concerning human rights and human rightlessness: disposability and struggle in the Bhopal gas disaster. *Third World Quarterly* 2015;36(6):1041-59.
25. Sathyamala C, Jayaprakash N. The Bhopal Disaster and Medical Research. *Economic and Political Weekly* 2012:37-40.
26. Khan I, Senthilkumar CS, Upadhyay N, et al. In silico docking of methyl isocyanate (MIC) and its hydrolytic product (1, 3-dimethylurea) shows significant interaction with DNA Methyltransferase 1 suggests cancer risk in Bhopal-Gas-Tragedy survivors. *Asian Pacific Journal of Cancer Prevention* 2015;16(17):7663-70.
27. Senthilkumar CS, Malla TM-u-D, Sah NK, et al. Cancer Morbidity Among Methyl Isocyanate Exposed Long-Term Survivors and Their Offspring: A Hospital-Based Five Year Descriptive Study (2006-2011) and Future Directions to Predict Cancer Risk in the Affected Population. *Asian Pacific journal of cancer prevention: APJCP* 2011;12(12):3443-52.
28. Senthilkumar CS, Akhter S, Malla TM, et al. Increased micronucleus frequency in peripheral blood lymphocytes contributes to cancer risk in the methyl isocyanate-affected population of Bhopal. *Asian Pac J Cancer Prev* 2015;16(10):4409-19. doi: 10.7314/apjcp.2015.16.10.4409 [published Online First: 2015/06/02]
29. National Centre for Disease Informatics and Research. Area Cancer National Cancer Registry Programme, India; 2021 [Available from: https://ncdirindia.org/Area_Cancer.aspx accessed June 8 2021.
30. Ministry of Social Justice and Empowerment, Department of Social Justice and Empowerment, Government of India. State-Wise Percentage of Population Below Poverty Line by Social Groups, 2004-05: Government of India; 2021 [Available from: http://socialjustice.nic.in/UserView/index?mid=76672 accessed June 8 2021.
31. Mehrotra S. Informal Employment Trends in the Indian Economy: Persistent informality, but growing positive development. In: Employment Policy Department I, ed. Employment: Working Paper No 254. Geneva, Switzerland: International Labour Organisation, 2019.
32. ILO Decent Work Team for South Asia and Country Office for India. India Wage Report: Wage policies for decent work and inclusive growth. Delhi, India: International Labour Organization, 2018.
33. Fulford S. Returns to education in India. *World Development* 2014;59:434-50.
34. Almond D, Mazumder B. Health Capital and the Prenatal Environment: The Effect of Ramadan Observance during Pregnancy. *American Economic Journal: Applied Economics* 2011;3(4):56-85. doi: 10.1257/app.3.4.56
35. Larsen AF, Headey D, Masters WA. Misreporting Month of Birth: Diagnosis and Implications for Research on Nutrition and Early Childhood in Developing Countries. *Demography* 2019;56(2):707-28. doi: 10.1007/s13524-018-0753-9
36. Malviya A, Ahirwar AK, Chandra Tripathi S, et al. COVID-19: a review on SARS-CoV-2 origin, epidemiology, virology, clinical manifestations and complications with special emphasis on adverse outcome in Bhopal Gas Tragedy survivor. *Horm Mol Biol Clin Investig* 2021;42(1):63-68. doi: 10.1515/hmbci-2020-0070 [published Online First: 2021/02/19]
37. Dore B. Covid-19: collateral damage of lockdown in India. *BMJ* 2020;369:m1711. doi: 10.1136/bmj.m1711 [published Online First: 2020/05/02]

For peer review only

For peer review only - http://bmjopen.bmj.com/site/about/guidelines.xhtml

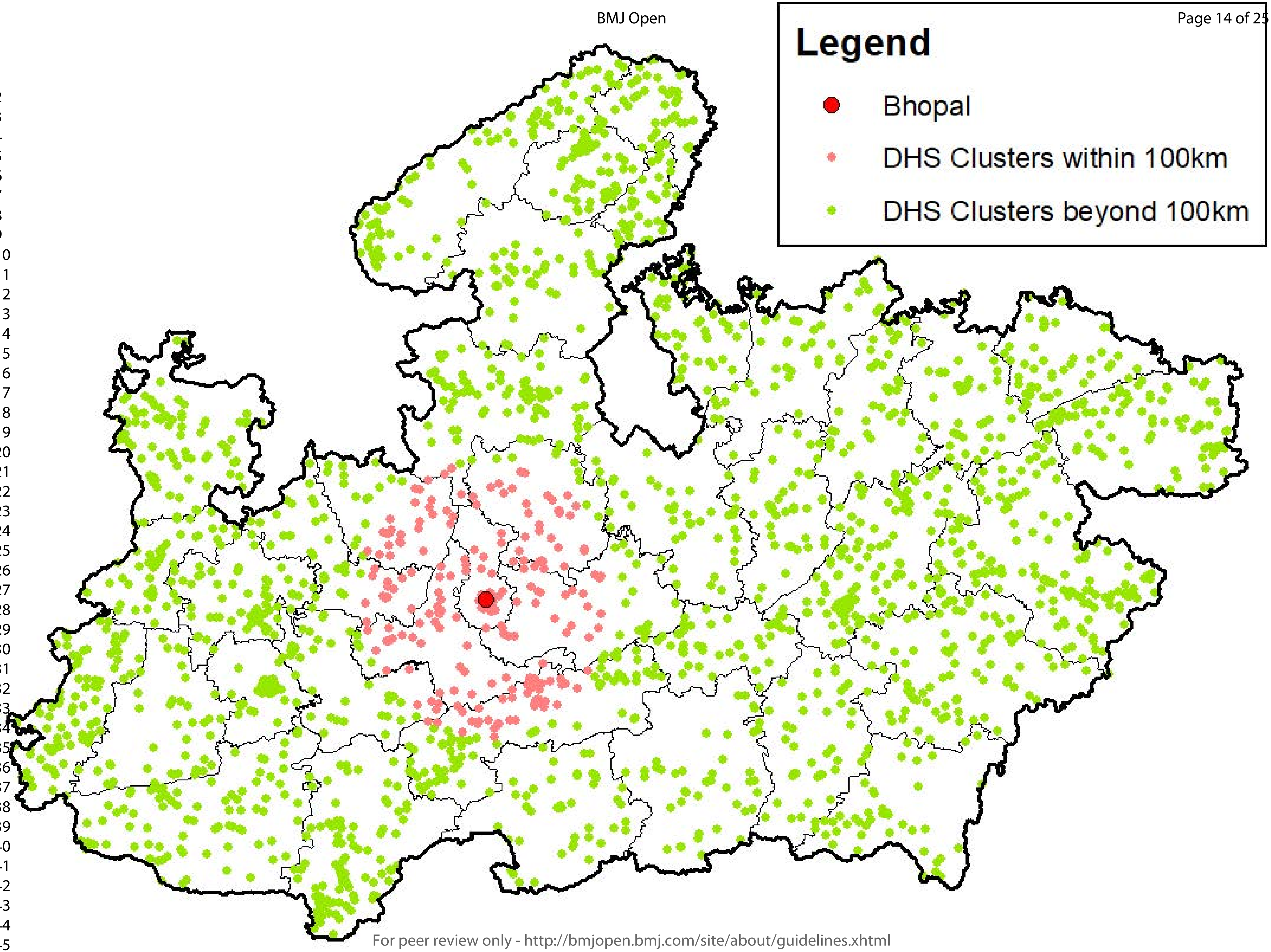

For peer review only - http://bmjopen.bmj.com/site/about/guidelines.xhtml

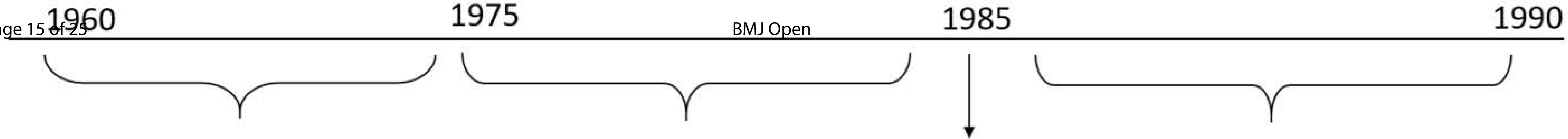

1960-1974	1975-1984	1985	1986-1990
Reference Cohort	T1	T2	T3
>10 years old at the time of Bhopal tragedy	0-10 years old at the time of Bhopal tragedy	Fetuses at the time of Bhopal tragedy	Not yet conceived at the time of Bhopal tragedy

For peer review only - http://bmjopen.bmj.com/site/about/guidelines.xhtml

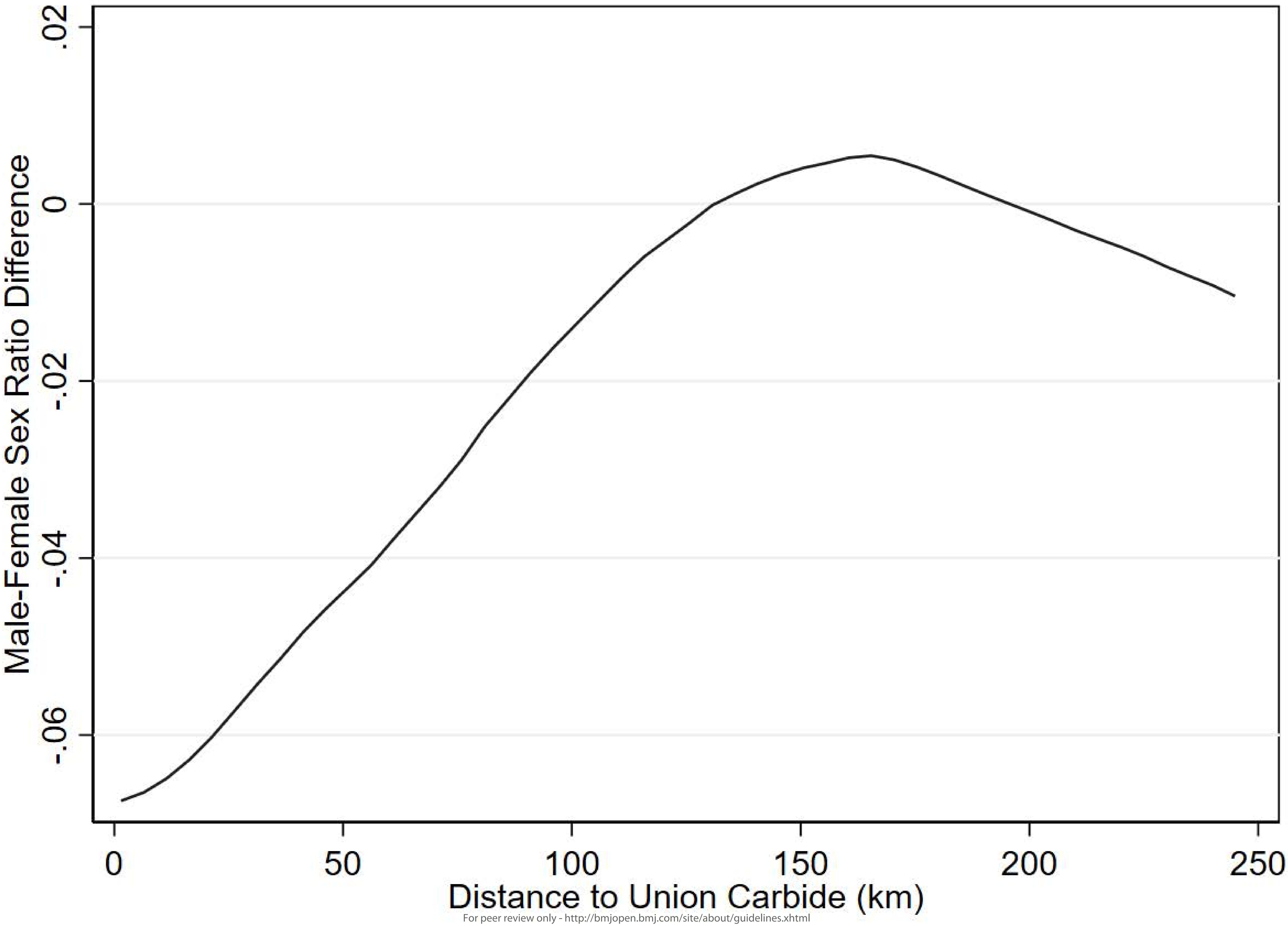
.02
0
-.02
-.04
-.06
Male-Female Sex Ratio Difference
0
50
100
150
200
250
Distance to Union Carbide (km)

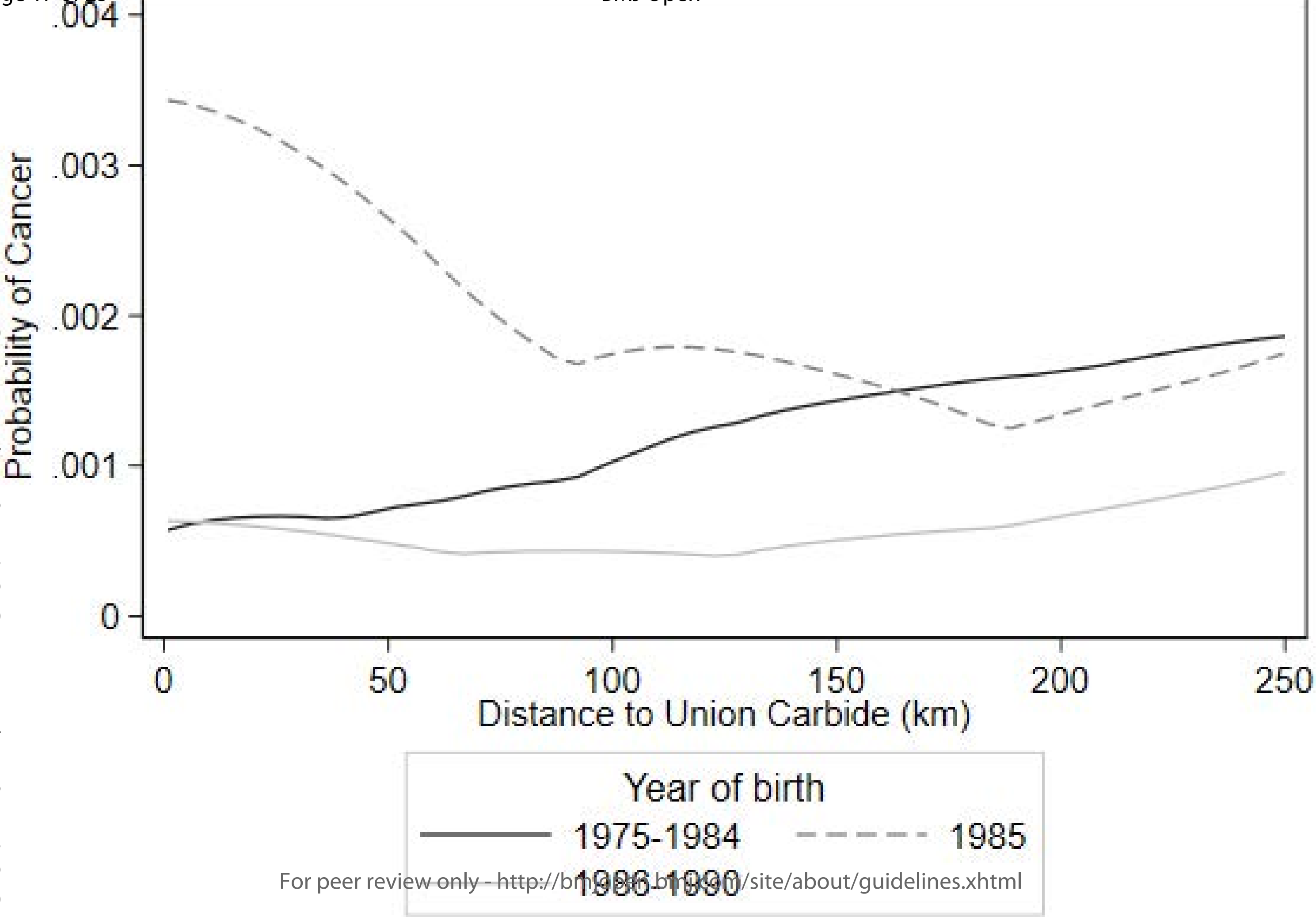
.004
.003
.002
.001
0
Probability of Cancer
0
50
100
150
200
250
Distance to Union Carbide (km)
Year of birth
1975-1984
1985

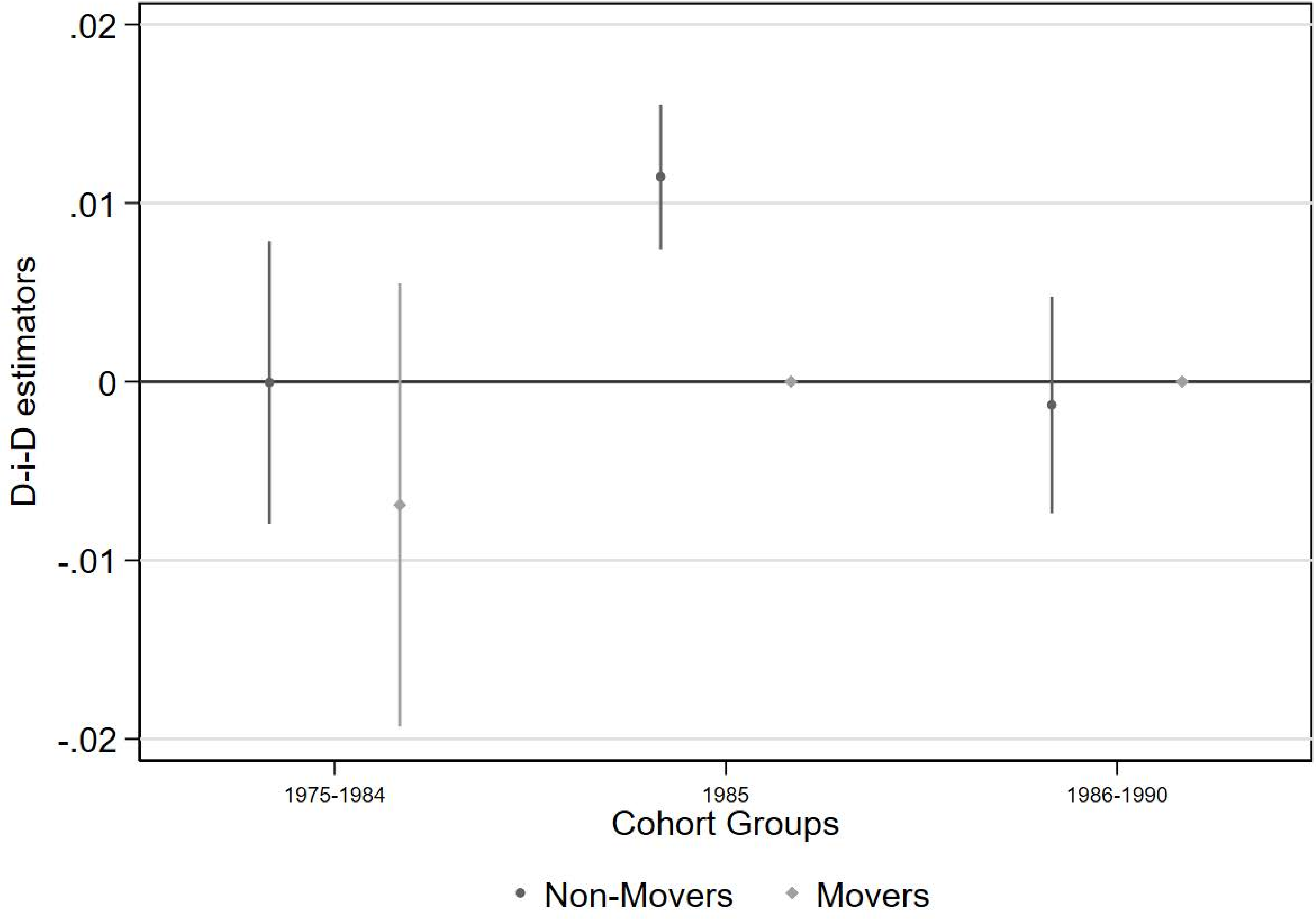
.02
.01
0
-.01
-.02
D-i-D estimators
1975-1984
1985
1986-1990
Cohort Groups
Non-Movers
Movers

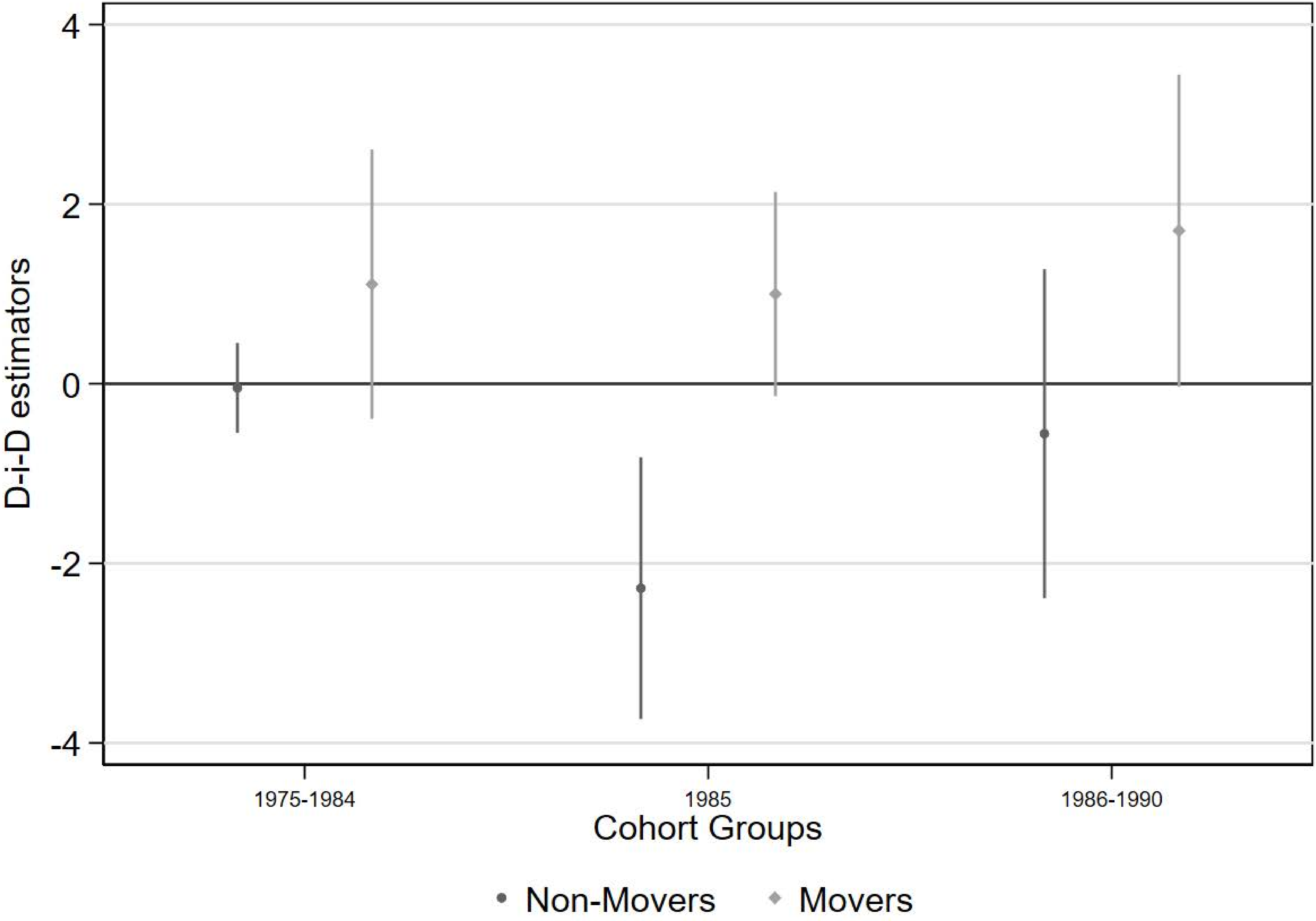
4
2
0
-2
-4
D-i-D estimators
1975-1984
1985
1986-1990
Cohort Groups
Non-Movers
Movers

In Utero Exposure to Industrial Disasters: A Case Study of the Bhopal Gas Tragedy

Supplemental Material

Gordon C. McCord[1]

Prashant Bharadwaj[2]

Lotus McDougal[3]

Arushi Kaushik[4]

Anita Raj[5]

Figure S1 uses the Indian Human Development Survey (IHDS) in a robustness check to test whether mothers of those who were in utero at the time of the BGD are different from those of other cohorts. The IHDS-I was conducted in 2004-05 and is representative at the district level. It is the only other survey apart from the DHS that collects health information for India. We compare mothers' education level and several (self-reported) health parameters, such as the probability of reporting to be suffering from a heart disease, high blood pressure, diabetes, asthma, tuberculosis, leprosy, cancer and mental illness (n=1,792).

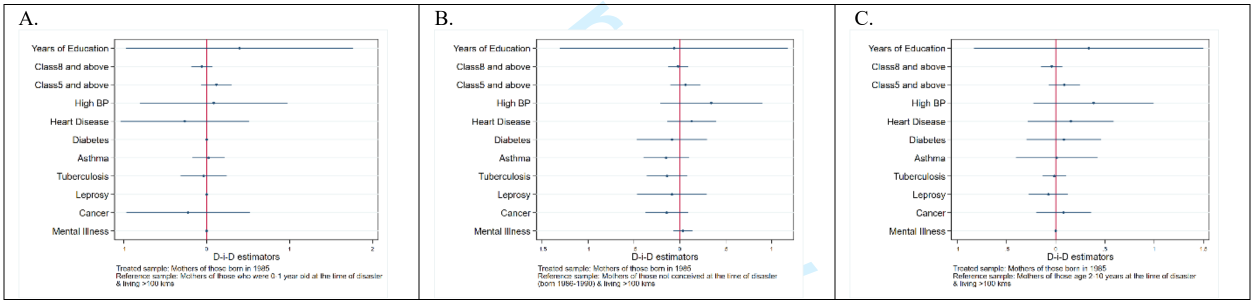

Figure S1: Characteristics of mothers of in utero cohort compared to others

The figure shows difference-in-difference estimates for various outcome variables for the mothers of those who were in utero (i.e, born in 1985). These estimates are obtained from regression equation similar to eq 1 where the control group includes mothers of non-mover men who were born between 1984 (i.e, < 1 year old at the time of BGD; panel A), non-mover men who were born between 1975-1983 (i.e, 2-10 years old at the time of BGD; panel B) and non-mover men who were born between 1986-1990 (i.e, not conceived at the time of BGD; panel C) and are living > 100kms away from Union Carbide plant. The figures depict estimates with 95% CI.

[1] Corresponding author. gmccord@ucsd.edu, School of Global Policy and Strategy, UC San Diego
[2] Department of Economics, UC San Diego
[3] Center on Gender Equity and Health, Department of Medicine, UC San Diego
[4] Department of Economics, UC San Diego
[5] Center on Gender Equity and Health, Department of Medicine, UC San Diego

For peer review only - http://bmjopen.bmj.com/site/about/guidelines.xhtml

Table S1: Linear probability models (LPM) for employment disability among men living in Madhya Pradesh in 1999 within 100km of Bhopal, compared to men of the same cohort more than 100km from Bhopal.

	Prob(Reported Employment Disability)		
	(1)	(2)	(3)
	Pooled	Non-Movers	Movers
1986-1990	-0.00109	-0.00131	0
	(0.00257)	(0.00297)	(5.63e-11)
1985	0.0111***	0.0115***	0
	(0.00216)	(0.00198)	(9.65e-11)
1975-1984	-0.000632	-4.65e-05	-0.00690
	(0.00344)	(0.00388)	(0.00608)
1960-1974	Reference	Reference	Reference
Observations	13,369	12,129	1,240
R-squared	0.003	0.003	0.024
Cohort FEs	Yes	Yes	Yes
Control Mean	0.00377	0.00426	0

Robust standard errors in parentheses

*** p<0.01, ** p<0.05, * p<0.1

Notes: The cohort of men born in 1985 within 100 km of Bhopal has a higher likelihood of reporting employment disability compared to other cohorts and to those born in Madhya Pradesh beyond 100 km from Bhopal (1), with effect most pronounced among men who have never moved (2) instead of those who have moved (3). Estimates are from ordinary least squares regressions as in Equation (I) with controls consisting of fixed effects for year of birth cohort and whether the DHS cluster is within 100 km of Bhopal. Standard errors are two-way clustered at the district and year-of-birth levels.

For peer review only

Table S2: Linear probability models (LPM) for employment disability among men who have never moved living in Madhya Pradesh in 1999 at various distances from Bhopal, compared to men of the same cohort more than 100km from Bhopal.

	Prob(Employment Disability) among Non-Movers, by Treatment Radius		
	(1)	(2)	(3)
	100 km	75 km	50 km
1986-1990	-0.00131	-0.00183	-0.00045
	(0.00297)	(0.00390)	(0.00445)
1985	0.0115***	0.0138***	0.0203**
	(0.00198)	(0.00217)	(0.0078)
1975-1984	-4.65e-05	-0.00049	-0.00004
	(0.00388)	(0.00314)	(0.00294)
1960-1974	Reference	Reference	Reference
Observations	12,129	11,799	11,100
R-squared	0.003	0.002	0.003
Cohort FEs	Yes	Yes	Yes
Control Mean	0.00426	0.00437	0.0043

Robust standard errors in parentheses
*** p<0.01, ** p<0.05, * p<0.1

Notes: Estimates are from ordinary least squares regressions as in Equation (I) with controls consisting of fixed effects for year of birth cohort and whether the DHS cluster is within the specified distance from Bhopal. Standard errors are two-way clustered at the district and year-of-birth levels.

For peer review only - http://bmjopen.bmj.com/site/about/guidelines.xhtml

Table S3: Regression results on educational attainment among men born in Madhya Pradesh within 100km of Bhopal, compared to men of the same cohort more than 100km from Bhopal.

	Years of Education Completed		
	(1)	(2)	(3)
	Pooled	Non-Movers	Movers
1986-1990	0.221	-0.557	1.704*
	(0.601)	(0.897)	(0.851)
1985	-0.577	-2.276***	0.999*
	(0.376)	(0.714)	(0.557)
1975-1984	0.255	-0.0471	1.108
	(0.279)	(0.245)	(0.734)
1960-1974	Reference	Reference	Reference
Observations	7,045	5,014	2,031
R-squared	0.048	0.055	0.047
Cohort FEs	Yes	Yes	Yes
Control Mean	5.833	5.561	6.600

Robust standard errors in parentheses

*** p<0.01, ** p<0.05, * p<0.1

Notes: The cohort of men born in 1985 within 100 km of Bhopal has fewer years of educational attainment compared to other cohorts and to those born in Madhya Pradesh beyond 100 km from Bhopal (1), with effect most pronounced among men who have never moved (2) instead of those who have moved (3). Estimates are from ordinary least squares regressions as in Equation (I) with controls consisting of fixed effects for year of birth cohort and whether the DHS cluster is within 100 km of Bhopal. Standard errors are two-way clustered at the district and year-of-birth levels.

For peer review only - http://bmjopen.bmj.com/site/about/guidelines.xhtml

Table S4: Regression results on educational attainment among non-mover men born in Madhya Pradesh at varying distances from Bhopal, compared to other men of the same cohort beyond 100 km from Bhopal.

	Years of Education Completed among Non-Movers, by Treatment Radius		
	(1)	(2)	(3)
	100 km	75 km	50 km
1986-1990	-0.557	-0.356	-0.264
	(0.897)	(0.682)	(0.454)
1985	-2.276***	-2.253**	-0.316
	(0.714)	(0.983)	(2.200)
1975-1984	-0.0471	-0.371	-0.900
	(0.245)	(1.292)	(1.970)
1960-1974	Reference	Reference	Reference
Observations	5,014	4,830	4,702
R-squared	0.055	0.055	0.053
Cohort FEs	Yes	Yes	Yes
Control Mean	5.561	5.584	5.643

Robust standard errors in parentheses

*** p<0.01, ** p<0.05, * p<0.1

Notes: Estimates are from ordinary least squares regressions as in Equation (I) with controls consisting of fixed effects for year of birth cohort and whether the DHS cluster is within the specified distance from Bhopal. Standard errors are two-way clustered at the district and year-of-birth levels.

For peer review only

For peer review only - http://bmjopen.bmj.com/site/about/guidelines.xhtml

STROBE Statement—Checklist of items that should be included in reports of ***cohort studies***

	Item No	Recommendation	Page No
**Title and abstract**	1	(*a*) Indicate the study's design with a commonly used term in the title or the abstract	1
		(*b*) Provide in the abstract an informative and balanced summary of what was done and what was found	1
**Introduction**			
Background/rationale	2	Explain the scientific background and rationale for the investigation being reported	2
Objectives	3	State specific objectives, including any prespecified hypotheses	3
**Methods**			
Study design	4	Present key elements of study design early in the paper	4
Setting	5	Describe the setting, locations, and relevant dates, including periods of recruitment, exposure, follow-up, and data collection	2-3
Participants	6	(*a*) Give the eligibility criteria, and the sources and methods of selection of participants. Describe methods of follow-up<br>(*b*) For matched studies, give matching criteria and number of exposed and unexposed	3
Variables	7	Clearly define all outcomes, exposures, predictors, potential confounders, and effect modifiers. Give diagnostic criteria, if applicable	3-4
Data sources/ measurement	8*	For each variable of interest, give sources of data and details of methods of assessment (measurement). Describe comparability of assessment methods if there is more than one group	3
Bias	9	Describe any efforts to address potential sources of bias	10
Study size	10	Explain how the study size was arrived at	3
Quantitative variables	11	Explain how quantitative variables were handled in the analyses. If applicable, describe which groupings were chosen and why	3
Statistical methods	12	(*a*) Describe all statistical methods, including those used to control for confounding<br>(*b*) Describe any methods used to examine subgroups and interactions<br>(*c*) Explain how missing data were addressed<br>(*d*) If applicable, explain how loss to follow-up was addressed<br>(*e*) Describe any sensitivity analyses	5
**Results**			
Participants	13*	(a) Report numbers of individuals at each stage of study—eg numbers potentially eligible, examined for eligibility, confirmed eligible, included in the study, completing follow-up, and analysed<br>(b) Give reasons for non-participation at each stage<br>(c) Consider use of a flow diagram	3
Descriptive data	14*	(a) Give characteristics of study participants (eg demographic, clinical, social) and information on exposures and potential confounders<br>(b) Indicate number of participants with missing data for each variable of interest<br>(c) Summarise follow-up time (eg, average and total amount)	3
Outcome data	15*	Report numbers of outcome events or summary measures over time	4

For peer review only - http://bmjopen.bmj.com/site/about/guidelines.xhtml

Main results	16	(*a*) Give unadjusted estimates and, if applicable, confounder-adjusted estimates and their precision (eg, 95% confidence interval). Make clear which confounders were adjusted for and why they were included<br>(*b*) Report category boundaries when continuous variables were categorized<br>(*c*) If relevant, consider translating estimates of relative risk into absolute risk for a meaningful time period	7
Other analyses	17	Report other analyses done—eg analyses of subgroups and interactions, and sensitivity analyses	7-9
**Discussion**			
Key results	18	Summarise key results with reference to study objectives	9
Limitations	19	Discuss limitations of the study, taking into account sources of potential bias or imprecision. Discuss both direction and magnitude of any potential bias	10
Interpretation	20	Give a cautious overall interpretation of results considering objectives, limitations, multiplicity of analyses, results from similar studies, and other relevant evidence	10
Generalisability	21	Discuss the generalisability (external validity) of the study results	10
**Other information**			
Funding	22	Give the source of funding and the role of the funders for the present study and, if applicable, for the original study on which the present article is based	1

*Give information separately for exposed and unexposed groups.

Note: An Explanation and Elaboration article discusses each checklist item and gives methodological background and published examples of transparent reporting. The STROBE checklist is best used in conjunction with this article (freely available on the Web sites of PLoS Medicine at http://www.plosmedicine.org/, Annals of Internal Medicine at http://www.annals.org/, and Epidemiology at http://www.epidem.com/). Information on the STROBE Initiative is available at http://www.strobe-statement.org.

For peer review only

For peer review only - http://bmjopen.bmj.com/site/about/guidelines.xhtml
